# Prevalence and seasonal dynamics of *bla*_CTX-M_ antibiotic resistance genes and fecal indicator organisms in the lower Lahn River, Germany

**DOI:** 10.1371/journal.pone.0232289

**Published:** 2020-04-30

**Authors:** Ilona Herrig, Susanne Fleischmann, Julia Regnery, Jessica Wesp, Georg Reifferscheid, Werner Manz

**Affiliations:** 1 Department G3 Biochemistry, Ecotoxicology, Federal Institute of Hydrology, Koblenz, Germany; 2 Department of Biology, Institute for Integrated Natural Sciences, University of Koblenz-Landau, Koblenz, Germany; Institut National de la Recherche Agronomique, FRANCE

## Abstract

Antibiotic-resistant bacteria represent an emerging global health problem and are frequently detected in riverine environments. Analyzing the occurrence of corresponding antibiotic-resistant genes in rivers is of public interest as it contributes towards understanding the origin and dissemination of these emerging microbial contaminants via surface water. This is critical for devising strategies to mitigate the spread of resistances in the environment. Concentrations of *bla*_CTX-M_ antibiotic resistance genes were quantified weekly over a 12-month period in Lahn River surface water at two sampling sites using quantitative real-time PCR. Gene abundances were statistically assessed with regard to previously determined concentrations of fecal indicator organisms *Escherichia coli*, intestinal enterococci and somatic coliphages, as well as influential environmental factors. Similar seasonal patterns and strong positive correlations between fecal indicators and *bla*_CTX-M_ genes indicated identical sources. Accordingly, linear regression analyses showed that *bla*_CTX-M_ concentrations could largely be explained by fecal pollution. *E*. *coli* provided the best estimates (75% explained variance) at the upstream site, where proportions of *bla*_CTX-M_ genes in relation to fecal indicator organisms were highest. At this site, rainfall proved to be more influential, hinting at surface runoff as an emission source. The level of agricultural impact increased from downstream to upstream, linking increasing *bla*_CTX-M_ concentrations after rainfall events to the degree of agricultural land use. Exposure assessment revealed that even participants in non-swimming recreational activities were at risk of incidentally ingesting *bla*_CTX-M_ genes and thus potentially antibiotic resistant bacteria. Considering that *bla*_CTX-M_ genes are ubiquitous in Lahn River and participants in bathing and non-bathing water sports are at risk of exposure, results highlight the importance of microbial water quality monitoring with an emphasis on antibiotic resistance not only in designated bathing waters. Moreover, *E*. *coli* might serve as a suitable estimate for the presence of respective antibiotic resistant strains.

## Introduction

Antibiotic-resistant bacteria (ARB) represent an emerging global health problem, which accounted for 67000 infections resulting in 33000 deaths in Europe in 2015 [1], and a further global increase is projected for the future [2]. Increasing resistance to 3^rd^ and 4^th^ generation cephalosporins in *Enterobacteriaceae* such as *Escherichia coli* is subject of growing concern [3–6]. Cephalosporin resistant *E*. *coli* belong to the ARB with the largest human health impact [1]. Resistance to cephalosporins, carbapenems and also penicillins in *Enterobacteriaceae* is mainly mediated by beta-lactam hydrolyzing enzymes (extended spectrum beta-lactamases, ESBL), of which CTX-M-type enzymes, encoded on plasmids by *bla*_CTX-M_ genes, are the most common (e. g. [7–9]). *E*. *coli* represents the dominant host of *bla*_CTX-M_ genes [9–11]. ESBL of the CTX-M-15 type are most commonly associated with clinical isolates from humans in Germany and other parts of the world [7–9].

ARB and their respective antibiotic resistance genes (ARG) including *bla*_CTX-M_ are increasingly detected outside clinical settings in various surface waters including rivers (e. g., [12–15]). They are released into aquatic environments from human and animal sources [16]. Studies emphasized that rivers comprise long distance transport and dispersal routes for ARB and ARG [17, 18] and pose transmission pathways to humans considering their manifold use, amongst others for recreational activities, drinking water abstraction, and crop irrigation. An increased risk of (re)transfer of ARB to humans through contact with surface water or wastewater was shown in previous studies (e. g. [19, 20]). As ARG proliferate among bacteria through horizontal gene transfer, human pathogens can acquire antibiotic resistance from non-pathogenic microorganisms and vice versa in natural environments under favorable conditions (e.g. [21, 22]). Although being recognized as a collecting vessel of antimicrobial resistance, knowledge about the factors governing the spread of ARB and ARG in the aquatic environment is still incomplete [23, 24]. Relative contributions of different sources and human health impacts caused by exposure to environmental resistant bacteria have also been identified as areas urgently in need of research [24].

Yet, ARG are important markers for tracking the spread of ARB in the environment and characterizing associated human health risks. In particular, *bla*_CTX-M_ genes that are frequently identified in *E*. *coli* as well as *E*. *coli* itself were suggested to be suitable to trace the dissemination of ARB in the environment [23, 25].

To gain more information on ARG prevalence and dynamics in effluent receiving surface waters and the contribution of fecal pollution sources, the relationship of *bla*_CTX-M_ gene and fecal indicator organism (FIO) concentrations (i.e., *E*. *coli*, intestinal enterococci, and somatic coliphages) and their mutual dependence on environmental driving factors were investigated at Lahn River, Germany. The two chosen riverine sampling sites are characterized by different degrees of wastewater effluent impact. Corresponding data of FIO and environmental parameters were obtained from a previous study by Herrig et al. [26].

Similar to other scenic rivers across Central Europe, Lahn River is very popular for waterborne recreation, especially boating and canoeing [27]. Thus, the potential exposure of recreational water users was assessed using water ingestion rates published in literature [28–30]. Furthermore, the ability of fecal indicator bacteria (FIB) to depict the dissemination of ARG was examined. As FIB concentrations are widely measured in routine monitoring schemes according to standardized protocols, the possibility of using standard FIB to monitor water quality with respect to antibiotic resistance would be extremely convenient for indicating risks of exposure.

## Materials and methods

### Study site

The study area is located at the lower stretch of Lahn River, a tributary of the Rhine River and is described in detail in Herrig et al. [26]. In brief, the scenic river is impounded by multiple weirs and locks and its mean annual discharge (MQ) is approximately 46.6 m^3^/s [31]. The study area is mostly surrounded by forested slopes, as well as narrow strips of meadows and pastures at the valley bottom. Agricultural areas are concentrated on the heights surrounding the valley; they are less prevalent in the river valley. Within the studied area, the degree of agricultural impact increases upriver, i.e., from sampling site 1 in the West (17% agricultural land use) to sampling site 2 in the East (36% agricultural land use) [32]. Although the lower Lahn valley is considered a rural environment, the proportion of municipal wastewater effluent at the studied river stretch is in the range of 10–20% during average flow conditions and greatly exceeds 50% under low flow conditions [33]. The river is predominantly used by smaller motor yachts, as well as paddle- and rowboats. Other recreational activities along this river stretch include fishing, canoeing, or water skiing [27].

Sampling site 1 (Lat 50.339052°N, Lon 7.681563°E) near the small town of Nievern (population of ~1000) is located approximately 1 km downstream of a municipal wastewater treatment plant (WWTP) outfall situated in the town of Bad Ems (population of ~9300). The WWTP with a treatment capacity of 33,000 person equivalents applies conventional treatment (i.e., tertiary treatment) and receives raw wastewater corresponding to 27,828 person equivalents from surrounding municipalities [34]. Sampling site 2 (Lat 50.316284°N, Lon 7.851037°E) is located 18 km upstream of sampling site 1 in the tiny town of Obernhof (population of ~375) and less impacted by municipal effluents. Upstream of sampling site 2, no immediate municipal WWTP outfalls or tributaries discharge into Lahn River over a stretch of approximately 9 km [26, 34]. Industrial dischargers within the study area comprise metalworking companies (approximately 15 km upstream of site 1 on the opposite shore) as well as a clinic (approximately 10 km upstream of site 1) [34].

### Collection of samples for molecular analyses

The collection of surface water grab samples for molecular analyses on a weekly basis (October 2011—December 2012) at both sampling sites was part of a broader monitoring campaign [26] at Lahn River. Samples were collected into sterile glass bottles from a depth of approximately 0.3 m below the water surface and 1 m off the shore using a telescopic stick. They were transported to the laboratory in an ice chest and were immediately processed upon arrival. Sample volumes of 100 mL to 250 mL (depending on suspended particle content) were filtered through cellulose acetate membrane filters with 0.2 μm pore size to retain bacteria for ARG analysis. The membrane filters with retained material were immediately placed in individual cryo tubes and were frozen at -80°C for further molecular analyses.

### DNA extraction and quantitative real-time PCR

Genomic DNA was extracted from the cellulose acetate filters using the DNeasy PowerWater DNA extraction Kit (Qiagen, Hilden, Germany) according to manufacturer’s instructions. Extracted DNA was kept at -20°C until further analysis (i.e., less than 3 months). Previous research demonstrated that freezing and extended storage of samples at -80°C does not alter ARG profiles compared to respective fresh samples [35].

SYBR Green qPCR was used to quantify *bla*_CTX-M_ genes encoding resistance to beta-lactam antibiotics and performed according to Marti et al. [36]. All qPCR assays were conducted on an Mx3005P system (Agilent Technologies, Santa Clara, USA). Each reaction was carried out in triplicate in a total volume of 30 μL, containing 20 μL of Brilliant III Ultra-Fast SYBR® Green QPCR Master Mix (Agilent Technologies), 3 μL of template DNA and forward and reverse primers (biomers, Ulm, Germany) in final concentrations of 300 nM for each primer. The final volume of 30 μL was completed with the respective amount of ultrapure water. Primers used (forward primer: CTATGGCACCACCAACGATA, reverse primer: ACGGCTTTCTGCCTTAGGTT) were originally published by Kim et al. [37] and modified by Marti et al. [36]. Cycling conditions consisted of one cycle at 95°C for 3 min followed by 45 cycles at 95°C for 15 s and 20 s at 60°C. To verify the specificity of PCR products, melting curve analyses were performed immediately after amplification in the range of 60°C to 95°C. Samples with melting curves indicative of unspecific products were excluded from further analyses if more than one replicate was affected. DNA of *E*. *coli* IMT 14355, which is known to harbor *bla*_CTX-M-3_ [38, 39], was obtained from the Institute of Microbiology and Epizootics (Freie Universität Berlin) and was analyzed as reference material.

Standard curves comprised 10-fold serial dilutions of *E*. *coli* IMT 14355 DNA in the range of 10 to 100,000 copies per reaction that were calculated according to the manufacturer’s protocol (Applied Biosystems, 2003) based on photometrically (Implen Nanophotometer P 330) determined reference DNA concentration (ng/μL). Calculation of the mass per genome was based on the *E*. *coli* median total genome length of 5.142 Mb [40].

The number of target gene copies per reaction was derived from the standard curves using the MxPro^™^ QPCR Software (Agilent Technologies). Gene copy number per 100 mL of sample volume was calculated according to Eq 1:

$$copies\;per\;100\;mL=\left(\frac{copies\;per\;reaction}{volume\;DNA\;per\;reaction}\right)x\left(\frac{volume\;of\;extracted\;DNA}{water\;sample\;volume}\right)x\;reference\;volume$$ (Eq 1)

Negative and positive controls were included in each run. Negative controls contained all the ingredients of the reaction mixture while template DNA was replaced by ultrapure water. Positive controls included DNA of *E*. *coli* IMT 14355.

### Fecal indicator organism data and environmental parameters

Corresponding spatiotemporal data of FIO abundances and general surface water characteristics for both sampling sites were retrieved from a previous investigation [26]. In brief, microbiological analyses had been conducted according to standard methods ISO 9308–3 [41], ISO 7899–2 [42], and ISO 10705–2 [43] as described in Herrig et al. [26]. FIO counts were expressed as MPN/100 mL (*E*. *coli*), CFU/100 mL (enterococci) and PFU/100 mL (coliphages).

General water characteristics including water temperature, specific conductivity, pH, turbidity, dissolved oxygen (O_2_) and chlorophyll-*a* had been measured *in situ* with a YSI 6600 V2 multiparameter sensor (YSI, USA) throughout the broader monitoring campaign [26]. Spectrophotometric measurements of nutrient concentrations (Xion 500, Hach-Lange, Germany) relied on ready-to-use cuvette tests (Hach-Lange, Germany). Global solar irradiance and precipitation data of nearby weather stations reported as daily totals and daily mean water discharge at gauge Kalkofen originated from the Rhineland-Palatinate rural area service center and the Federal Institute of Hydrology, respectively [26].

### Statistical analyses

All statistical analyses were performed using the statistical software R [44]. Individual Spearman’s rank correlations as well as principal component analyses (PCA) were run to identify relationships between gene and indicator concentrations and environmental parameters as well as for identification of seasonal patterns. Assignment of seasons (spring, summer, fall, winter) followed the astronomical beginning of seasons for the Central European Time Zone (UTC+1). Samples containing ARG concentrations below the limit of quantification (LOQ) of 10 copies per reaction were excluded from statistical analyses. PCA and Spearman’s rank correlations were performed on z-standardized data. FIO concentrations and ARG concentrations were log_10_ transformed and linear regression was performed on the whole dataset including concentration data pooled of both sites using the *lm()* function in R. ARG concentrations in surface water were predicted individually for each site on the basis of *E*. *coli*, intestinal enterococci and somatic coliphages by the linear model using the function *predict()* in R.

### Exposure and risk assessment

Assuming that 32% to 48% [45] of *E*. *coli* detected in freshwater are antibiotic-resistant, theoretical minimum, average and maximum concentrations of resistant *E*. *coli* in Lahn River were calculated, based on minimum, average and maximum *E*. *coli* concentrations measured in Lahn during the study period [26]. Similar calculations were performed to estimate minimum, average and maximum theoretical concentrations of ESBL-producing *E*. *coli* in Lahn River, assuming that ESBL-producing *E*. *coli* represent 0.05% [46] to 1.7% [47] of total *E*. *coli* in freshwater. Taking into account that 8.5% of all ESBL-producing *E*. *coli* in surface water can be suspected gastrointestinal pathogens [48], theoretical minimum, average and maximum numbers of ESBL-producing diarrheagenic *E*. *coli* in Lahn River were also calculated. Subsequently, human water sports related exposure was estimated for total *E*. *coli*, antibiotic-resistant *E*. *coli*, ESBL-producing *E*. *coli*, diarrheagenic ESBL-producing *E*. *coli* as well as for *bla*_CTX-M_ genes using ingestion rates from literature [28–30]. Ingestion rates of bacteria and genes per hour as well as bacteria and genes per water sport session were calculated based on data about average times people spend at particular water sports. Exposure assessment was conducted for non-swimming water sports including boating, fishing, rowing, canoeing, kayaking [29, 30], and for swimming [28]. Dufour et al. [29] reported mean water ingestion rates during active swimming in a swimming pool of 16 mL/45 min (49.33 mL/h) for adults and 37 mL/45 min (21.33 mL/h) for children. It was suggested that these volumes may also apply to swimming in freshwater. Schets et al. [49] determined by questionnaires that visits at freshwater sites lasted up to 79 minutes (1.32 h). The extent to which participants in boating, fishing and canoeing may be exposed via incidental ingestion of water was estimated based on median ingestion rates (50^th^ percentile) published by Rijal et al. [30]. Dorevitch et al. [28] reported ingestion rates for boating, canoeing, fishing, rowing and kayaking of 3.7 mL, 3.9 mL, 3.6 mL, 3.5 mL, and 3.8 mL, respectively. For reasons of comparability, ingestion rates for boating, canoeing and fishing by Dorevitch et al. [28] were used together with the respective durations specified by Rijal et al. [30] (i.e. 4 h for boating and fishing, 2.6 h for canoeing).

Risk assessment was conducted using established beta-Poisson dose–response models, as shown in equation (Eq 2) [50], to calculate the probability of infection after exposure to ESBL-carrying diarrheagenic *E*. *coli*.

$$P_{\left(response\right)}=1-{\left[1+dose\frac{\left(2^{\frac{1}{\alpha }}-1\right)}{N_{50}}\right]}^{-\alpha }$$ (Eq 2)

P_(response)_ is the probability of infection, dose = c*V (where *c* is the assumed concentration of hazards in the water and *V* is the volume of water ingested). Median infectious doses (N_50_) and slope parameters (alpha) were obtained from Haas et al. [50] and DuPont et al. [51].

## Results and discussion

### Prevalence of *bla*_CTX-M_ genes and fecal indicators

ARG prevalence and dynamics were examined in order to assess the potential exposure of water sports participants at Lahn River and the ability of fecal indicator bacteria (FIB) to depict the dissemination of ARG. In accordance with their spatial proximity, both sampling sites at Lahn River were very similar in terms of their hydrological, hydrochemical and meteorological characteristics throughout the study period from October 2011 until October 2012 (Table 1).

**Table 1:** Overview of parameters measured during the sampling period (adapted from Herrig et al. [26]).

		site 1						site 2					
parameter	unit	median	min	max	average	SD	n (n below LOQ)	median	min	max	average	SD	n (n below LOQ)
***bla***_**CTX-M**_	**[copies/100 mL]**	1630	349	12320	2905	3097	33 (13)	1454	412	20083	3453	4640	45 (6)
***E*. *coli***	**[MPN/100 mL]**	1579	212	27730	3571	5296	63	596	15	23670	2745	5105	64
**enterococci**	**[CFU/ 100 mL]**	252	75	10150	665	1417	60	112	3	11450	621	1622	61
**coliphages**	**[PFU/100 mL]**	1010	120	6760	1492	1539	61	615	60	8550	1531	1986	62
**discharge**	**[m^2^/s]**	19	10	248	34	45	65	19	10	248	34	45	65
**water temperature**	**[°C]**	10.2	1.1	22.6	11.3	5.9	61	9.9	0.2	22.4	11.1	5.9	61
**conductivity**	**[**μ**S/cm]**	452	231	607	447	96	61	450	232	608	447	92	61
**pH**	**[–]**	8.2	7.0	8.7	8.1	0.3	61	8.1	7.2	8.9	8.1	0.3	61
**turbidity**	**[NTU]**	3.5	0.9	69.4	8.5	12.5	61	3.2	1.0	75.9	7.6	13.9	61
**chlorophyll-*a***	**[**μ**g/L]**	5.1	1.2	69.2	10.3	13.5	61	4.3	0.7	68.7	9.7	13.4	61
**oxygen**	**[mg/L]**	11.1	8.2	14.3	11.0	1.7	61	10.8	6.7	14.7	10.9	2.2	61
**rainfall**	**[mm]**	0.2	0.0	15.0	1.9	3.3	65	0.2	0.0	15.0	1.9	3.3	65
**rainfall**_**(4d-sum)**_	**[mm]**	5.8	0.0	36.4	7.8	7.6	64	5.8	0.0	36.4	7.8	7.6	64
**NO**_**2**_**-N**	**[mg/L]**	0.03	0.01	0.07	0.03	0.01	53	0.03	0.01	0.10	0.03	0.02	54
**NO**_**3**_**-N**	**[mg/L]**	2.72	1.85	5.46	2.75	0.59	53	2.75	1.89	5.69	2.82	0.63	53
**NH**_**4**_**-N**	**[mg/L]**	0.06	0.01	0.43	0.08	0.07	53	0.05	0.01	0.51	0.09	0.09	53
**PO**_**4**_**-P**	**[mg/L]**	0.23	0.02	0.55	0.23	0.07	51	0.24	0.12	0.55	0.24	0.07	50
**TN**_**b**_	**[mg/L]**	3.28	1.62	7.44	3.36	0.78	50	3.41	2.61	6.39	3.46	0.67	50
**global solar irradiance**	**[Wh/m^2^]**	1925	177	7940	2692	2207	65	1925	177	7940	2692	2207	65
**global solar irradiance**_**(3d-sum)**_	**[Wh/m^2^]**	6655	593	22395	8322	5982	65	6655	593	22395	8322	5982	65

SD: standard deviation; min: minimum; max: maximum; n: number of observations; TN_b_: total nitrogen bound; MPN: most probable number; CFU: colony forming units; PFU: plaque forming units.

**Fig 1:**
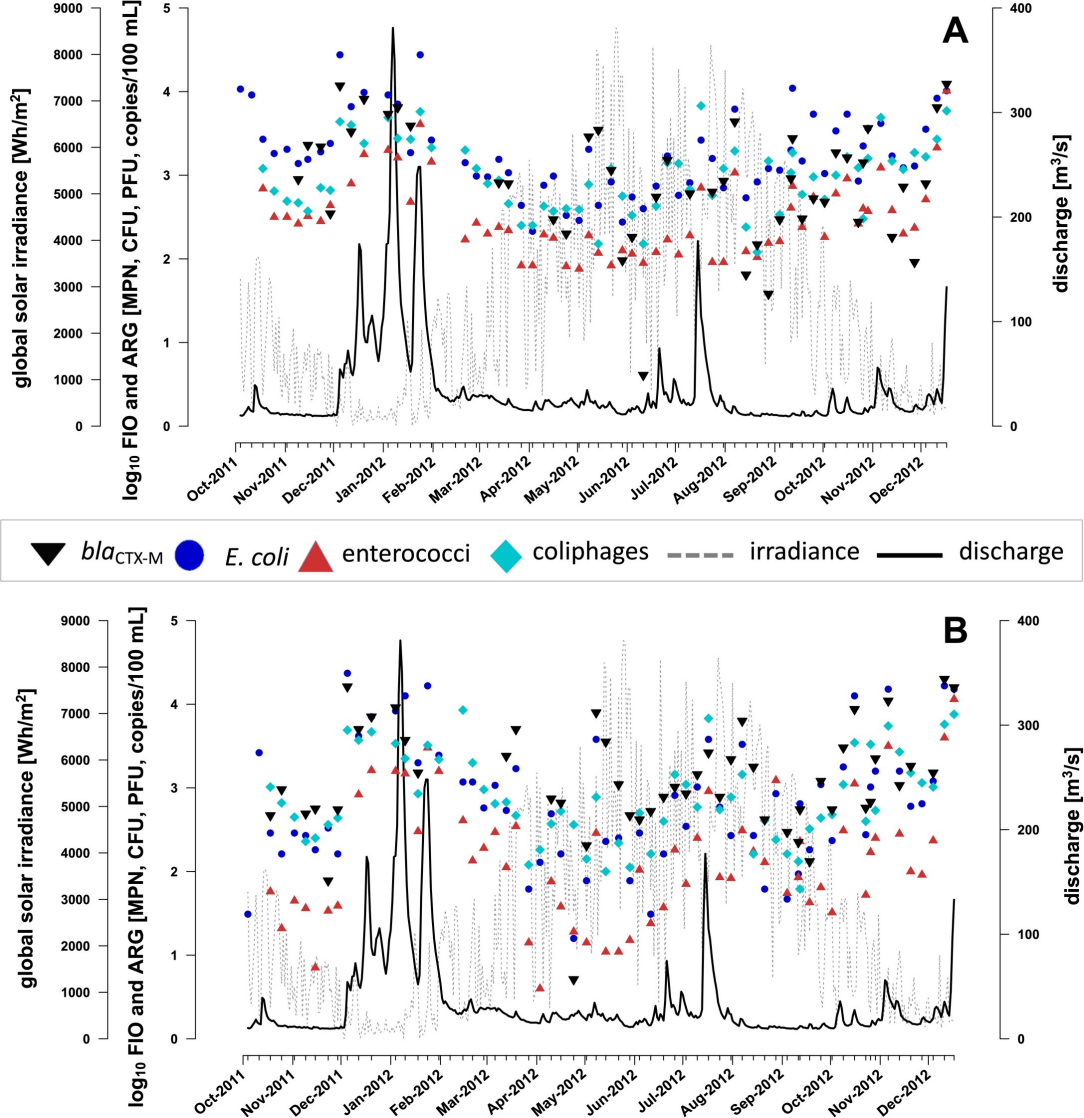
Distribution of *bla*_CTX-M_ genes and FIO in Lahn River throughout the study period. Log_10_-transformed concentrations of FIO *E*. *coli*, intestinal enterococci, somatic coliphages and *bla*_CTX-M_ genes (A) at site 1 and (B) at site 2 are shown in relation to global solar irradiance and discharge.

The river’s daily mean discharge ranged between a minimum of 9 m^3^/s in September 2012 (low flow conditions) and a maximum of 381 m^3^/s in January 2012 (high flow conditions) (Fig 1). Sampling events covered daily mean discharge conditions that varied between 10 and 248 m^3^/s, respectively (Table 1).

FIO [26] and *bla*_CTX-M_ ARG were detected in all water samples at both sites throughout the year. *E*. *coli* exhibited highest average FIO concentrations, whereas average enterococci concentrations were lowest. Measured *bla*_CTX-M_ concentrations ranged between 3.49 x 10^2^ and 2.01 x 10^4^ copies/100 mL (Table 1).

**Fig 2:**
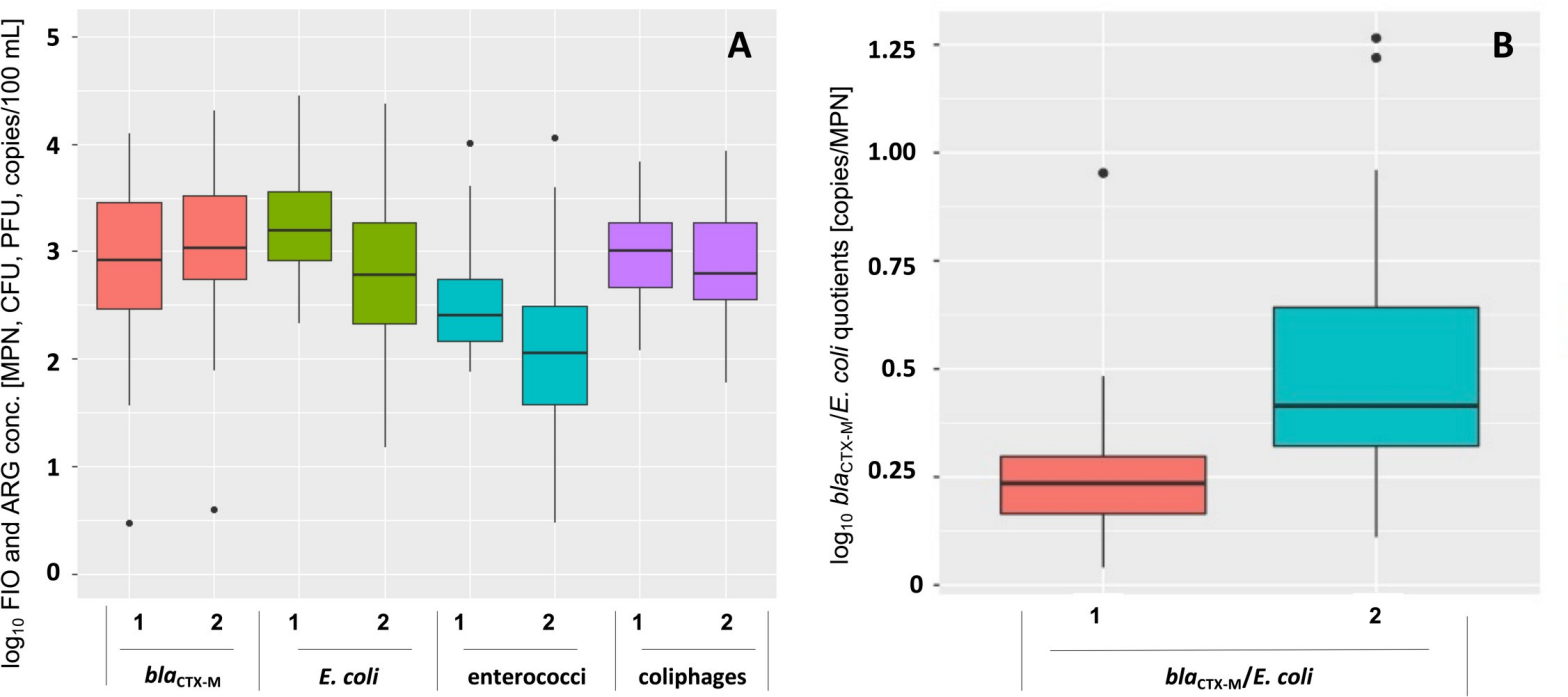
Boxplots of *bla*_CTX-M_ and FIO concentrations in Lahn River surface water samples throughout the sampling period at sites 1 and 2. (A) Concentrations of total annual FIO and *bla*_CTX-M_ genes. (B) Concentrations of annual *bla*_CTX-M_ genes normalized to *E*. *coli* concentrations.

The coefficients of determination (R^2^) of standard curves in qPCR experiments ranged from 0.994 to 0.999. Efficiencies between 90.3% and 92.3% were obtained over at least 5 orders of magnitude in all qPCR runs, confirming the validity of the assay. At sampling site 1 in Nievern, 13 out of 46 observations of *bla*_CTX-M_ genes were below the LOQ of 10 copies per reaction. At sampling site 2 in Obernhof, only 6 out of 51 analyzed samples were below LOQ. In total, annual *bla*_CTX-M_ concentrations did not differ significantly between both sites (Fig 2A). At site 2, relative proportions of ARG expressed as the quotient of ARG and *E*. *coli* were elevated compared to site 1 (Fig 2B).

### Relations between ARG, fecal indicators and environmental parameters

PCA and individual Spearman’s rank correlations revealed relations between ARG, FIO and environmental data, as well as seasonal patterns. ARG correlated with FIO, especially *E*. *coli* (Table 2) and the ARG distribution pattern closely resembled that of *E*. *coli* over the year (Fig 1). This is an indication that ARG and *E*. *coli* originate from the same pollution sources and are distributed within the river in similar ways. Interestingly, *bla*_CTX-M_ genes correlated strongest with FIB concentrations at site 2 (Table 2). The strong correlations between *bla*_CTX-M_ and *E*. *coli* corroborate current findings of other studies [52, 53] and reflect that *E*. *coli* is the dominant host of *bla*_CTX-M_ genes [10, 11].

**Table 2:** Individual Spearman’s rank correlations between the FIO and *bla*_CTX-M_ gene concentrations and environmental parameters.

	site 1 (n = 25)				site 2 (n = 35)			
	*bla*_CTX-M_	*E*. *coli*	enterococci	coliphages	*bla*_CTX-M_	*E*. *coli*	enterococci	coliphages
*bla*_CTX-M_		**0.76**	**0.71**	**0.78**		**0.88**	**0.81**	**0.73**
*E*. *coli*	**0.76**		**0.91**	**0.75**	**0.88**		**0.93**	**0.79**
enterococci	**0.71**	**0.91**		**0.71**	**0.81**	**0.93**		**0.78**
coliphages	**0.78**	**0.75**	**0.71**		**0.73**	**0.79**	**0.78**	** **
discharge	**0.55**	0.29	0.29	**0.50**	**0.69**	**0.65**	**0.66**	**0.72**
water temperature	**-0.43**	**-0.47**	**-0.56**	**-0.43**	**-0.58**	**-0.60**	**-0.53**	**-0.54**
conductivity	-0.17	0.11	0.11	-0.12	**-0.42**	-0.30	-0.30	**-0.49**
pH	-0.22	-0.19	-0.31	-0.09	**-0.40**	**-0.41**	**-0.41**	**-0.43**
turbidity	**0.58**	0.38	0.32	**0.65**	0.30	0.29	0.33	**0.44**
chlorophyll-*a*	0.05	-0.24	-0.25	-0.09	-0.19	**-0.34**	-0.26	-0.32
dissolved oxygen	**0.48**	0.36	**0.44**	**0.43**	**0.53**	**0.52**	**0.47**	**0.46**
rainfall	0.13	0.33	0.28	0.03	-0.01	0.14	0.12	0.03
rainfall_(4d-sum)_	**0.69**	**0.56**	**0.58**	**0.54**	**0.56**	**0.65**	**0.64**	**0.52**
NO_2_-N	**0.54**	0.36	0.35	0.55	**0.42**	**0.45**	**0.42**	**0.53**
NO_3_-N	0.35	**0.50**	**0.64**	0.34	0.33	**0.41**	**0.42**	**0.35**
NH_4_-N	**0.69**	**0.73**	0.73	0.62	**0.61**	**0.74**	**0.69**	**0.64**
PO_4_-P	0.07	0.08	0.02	-0.05	0.01	0.07	0.03	-0.03
TN_b_	0.17	**0.42**	**0.55**	0.26	**0.44**	**0.50**	**0.47**	**0.43**
global solar irradiance	**-0.43**	**-0.65**	**-0.77**	-0.51	**-0.44**	**-0.55**	**-0.51**	**-0.48**
global solar irradiance_(3d-sum)_	**-0.51**	**-0.68**	**-0.78**	-0.57	**-0.58**	**-0.72**	**-0.66**	**-0.62**

Color gradient indicates strength of correlation with positive correlations in blue, negative correlations in purple, significant (p<0.05) correlations in bold.

All measured environmental parameters except PO_4_-P correlated with at least one FIO or ARG and can thus be considered as potentially relevant for their fate and transport. Levels of FIO and ARG increased with discharge, turbidity, dissolved oxygen, rainfall, and nutrient concentrations, whereas water temperature, conductivity, pH, chlorophyll-*a*, and global solar irradiance were associated with a decline in FIO and ARG concentrations (Table 2).

**Fig 3:**
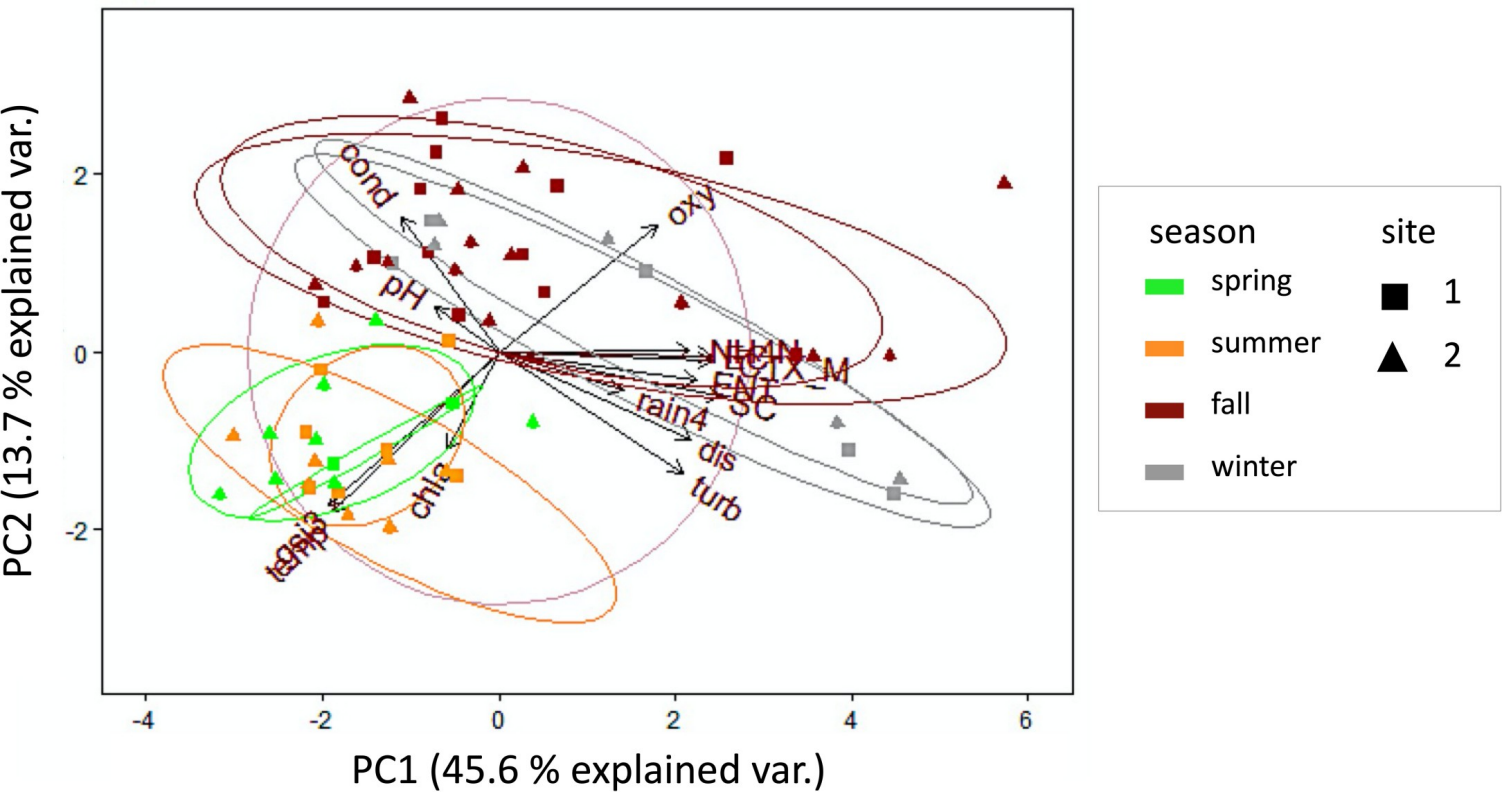
PCA biplot of *bla*_CTX-M_ genes, FIO and environmental parameters. Site 1 data are indicated by squares, site 2 data are indicated by triangles. Data are grouped by seasons (cellipses); (circle = correlation circle, chla: chlorophyll-*a*, cond: conductivity, CTX_M: *bla*_CTX-M_ genes, dis: discharge, EC: *E*. *coli*, ENT: intestinal enterococci, gsi3: 3-day-sum of global solar irradiance, NH4N: ammonium-nitrogen, oxy: oxygen, rain4: 4-day-sum of rainfall, SC: somatic coliphages, temp: water temperature, turb: turbidity).

Accordingly, *bla*_CTX-M_ concentrations showed strong seasonal alterations and varied over nearly 2 orders of magnitude (Table 1, Fig 1). High concentrations of FIO and ARG were measured predominantly during fall and winter. Fall and winter were characterized by high discharge following rainfall events, elevated oxygen levels due to cold water temperatures, elevated turbidity due to resuspension and runoff, and rising NH_4_-N contents, indicating an influence of wastewater discharges. During spring and summer FIO and ARG concentrations declined (Fig 1, Fig 3). Both seasons were characterized by increasing global solar irradiance and therefore accompanied by rising water temperature and chlorophyll-*a* levels.

**Fig 4:**
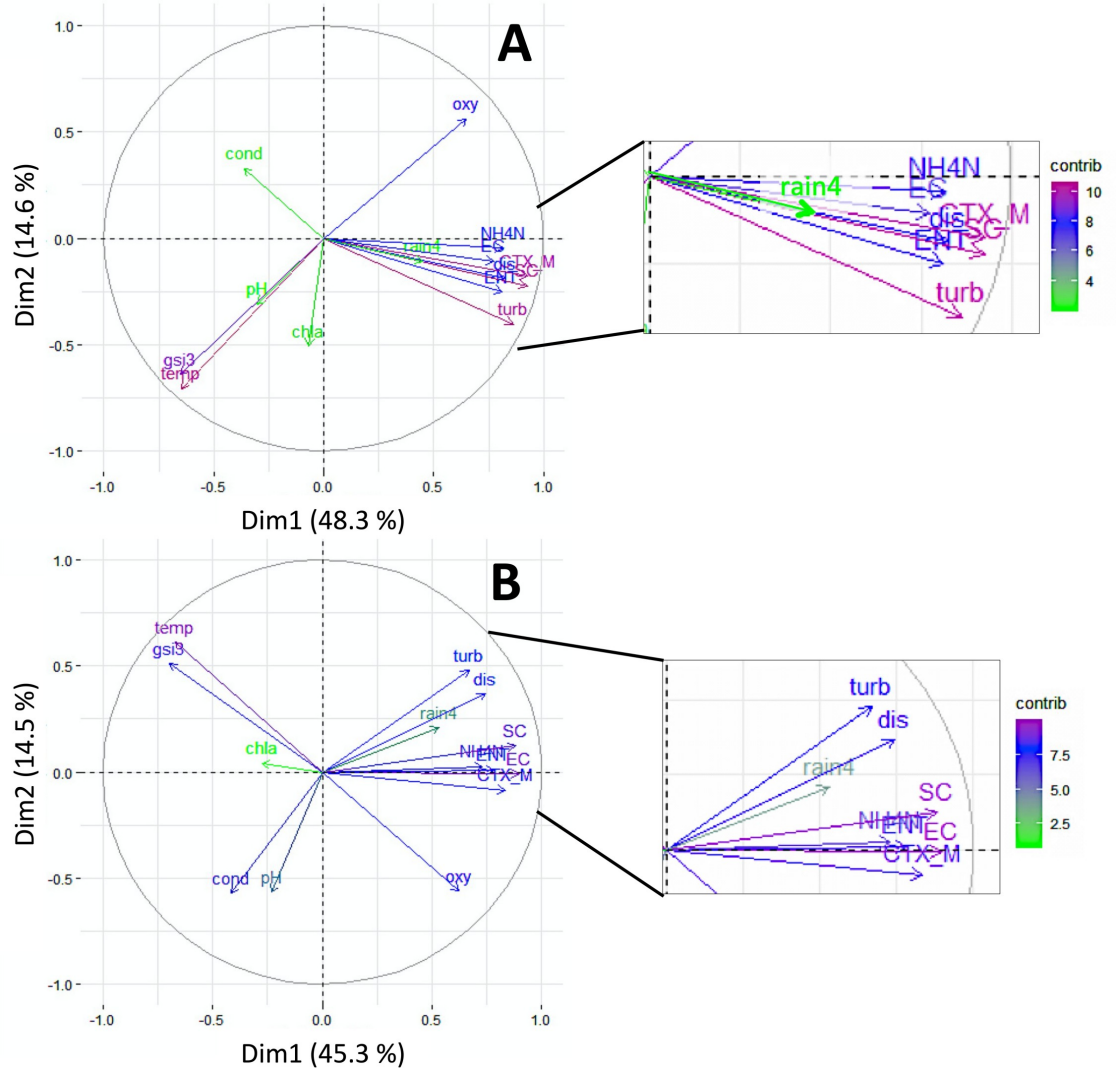
PCA biplot of *bla*_CTX-M_ genes, FIO and environmental parameters with correlation circle and contributions indicated by color gradient. Data are shown individually for (A) site 1 and (B) site 2 (Abbreviations: rain4: 4-day-sum of rainfall; temp: water temperature; cond: conductivity; gsi3: 3-day-sum of global solar irradiance; chla: chlorophyll-*a*; dis: discharge; CTX_M: bla_CTX_M_ genes; EC: *E*. *coli*; ENT: intestinal enterococci; SC: somatic coliphages; turb: turbidity; oxy: oxygen content; NH4N: ammonium-nitrogen; contrib: contribution).

Water temperature, global solar irradiance (3d-sum), turbidity, NH_4_-N and discharge were environmental parameters that contributed most to explained variance in PCA (Fig 3). Similar relationships had been demonstrated for FIO concentrations at rivers Rhine and Moselle and dependencies between environmental parameters and FIO were extensively discussed in previous studies [26, 54]. A comparison of PCA results between sites 1 and 2 showed similar contributions and comparable proportions of explained variance at both sites. However, it is notable that rainfall, pH, and conductivity contributed to a greater extent to the explained variance of site 2 data (Fig 4).

**Fig 5:**
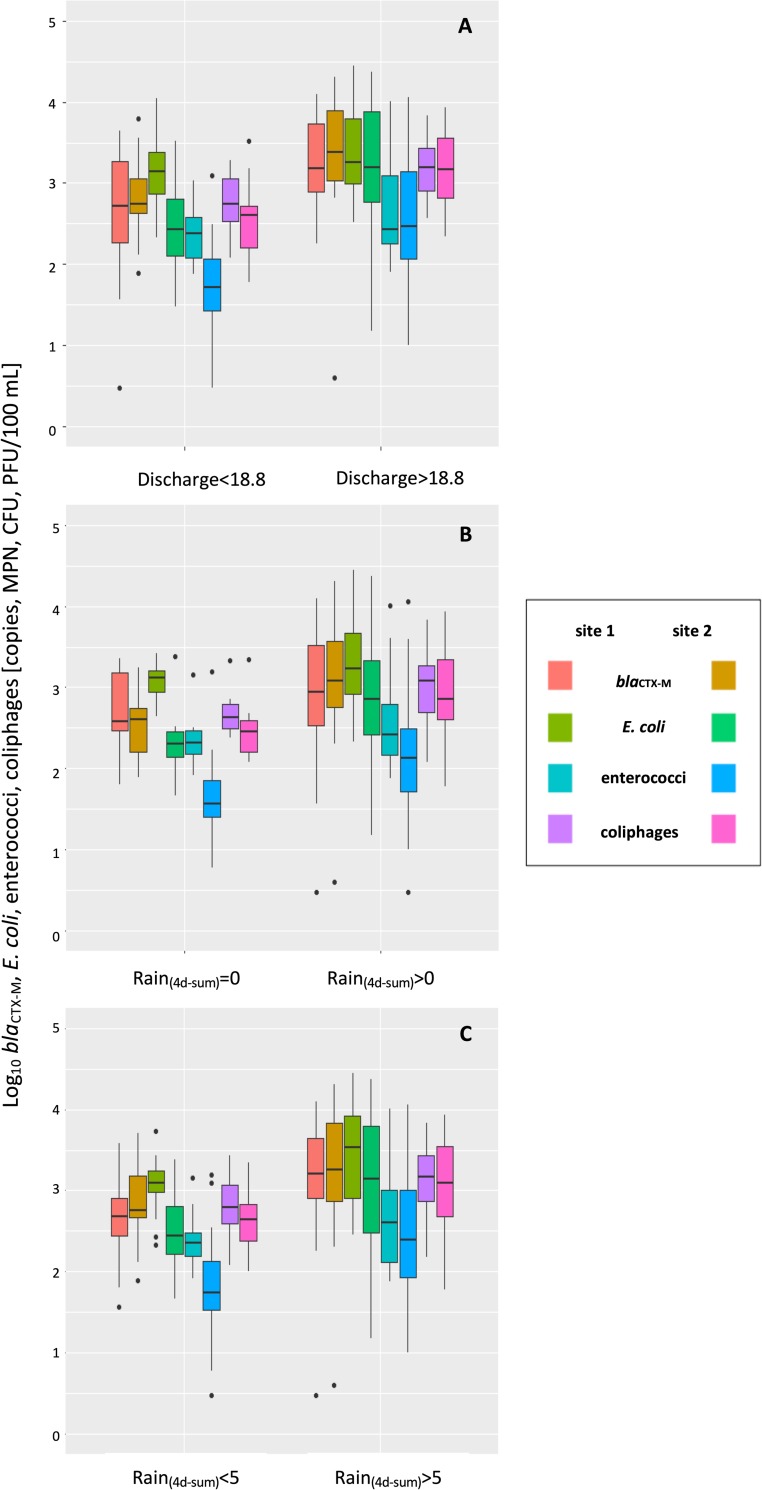
Boxplots of fecal indicator and ARG concentrations. **(**A) low flow periods (discharge < median of 18.8 m^3^/s) versus high flow periods (discharge > median of 18.8 m^3^/s) (B) in dry periods (4d-sum of rainfall = 0 mm) versus wet periods (4d-sum of rainfall > 0 mm) and (C) in periods with low rainfall (4d-sum of rainfall < 5mm) versus high rainfall (4d-sum of rainfall > 5 mm).

A strong impact of rainfall and related discharge especially at site 2 is also shown in Fig 5.

During high flow periods, annual FIO and *bla*_CTX-M_ levels were comparable between both sampling sites (Fig 5A). During periods without rainfall 4 days prior sampling, FIO and *bla*_CTX-M_ concentrations tended to be higher at site 1 (Fig 5B). If precipitation was higher than 5 mm over the four-day period prior sampling, *bla*_CTX-M_ concentrations at site 2 exceeded those measured at site 1 (Fig 5C), indicating a more prominent influence of surface runoff related emissions at site 2.

### Impact of point and non-point sources

It is well known that ARB and ARG are released into the environment from various sources including wastewater discharges and agriculture (e.g. [55]) and studies found WWTPs to significantly enhance ARG levels and diversity in rivers [56, 57] including *bla*_CTX-M_ [36, 58]. A strong correlation with NH_4_-N indicates an influence of wastewater on elevated levels of FIO and ARG. But despite the close proximity of site 1 to the WWTP outfall in Bad Ems, no direct influence of the municipal WWTP on elevated annual total ARG levels was observed (Fig 2A). Yet, an influence of the WWTP is illustrated by decreased relative percentages of *bla*_CTX_M_ genes compared to *E*. *coli* at site 1 (Fig 2B). Similar observations were made by Haberecht et al. [47], where percentages of *E*. *coli* harboring ESBL resistance were lower in WWTP effluent (0.28%) compared to surface water (1.7%). To depict the impact of individual wastewater contributions with regard to ARG levels in rivers, dry-season sampling proved to be useful [59]. If dry- and wet-weather data are assessed separately, the impact of WWTP discharges becomes apparent at site 1 during periods without rainfall events and low discharge (Fig 1, Fig 5). Without input of pollutants by rainfall and surface runoff, microbiological determinants substantially declined at site 2 during low flow periods between May and June, whereas concentrations remained elevated at site 1 (Fig 1, Fig 5B). Clearly, the WWTP provided a constant input of microbial pollutants at sampling site 1. However, other inputs of fecal pollution exceeding that of the municipal WWTP mask the constant effluent-related background pollution during high flow conditions. At site 2, rainfall events likely promote a flush of (suspended) particulate matter in runoff from surrounding agricultural areas into the river, explaining the higher impact of rainfall at this site. ARG pollution increasing with anthropogenic or agricultural impact was also described for other aquatic environments [60–62]. Interestingly, rainfall at site 2 impacted FIO abundances to a lesser extent than *bla*_CTX-M_ gene abundances (Fig 5C). This may be due to different detection rates, persistence and transport properties of DNA and living cells. As ARG quantified by qPCR may comprise extracellular DNA (eDNA) (which may be retained on the cellulose acetate filters, when attached to particles) and intracellular DNA, ARG are likely to be detected in higher concentrations than living cells of FIO quantified by cultivation dependent methods.

eDNA is known to adsorb to soil components including clay, sand, silt and humic substances [63], protecting it against degradation. This can considerably prolong its persistence in soil and sediments, which may even facilitate ARG propagation [63–65]. It is hypothesized that DNA may be detectable for a longer time and therefore longer distances compared to living cells due to its different persistence properties and adsorption to particles. Transport of DNA including ARG through soil is known to occur [66–68]. Even an on-site selection due to antibiotic residues in the environment, leading to elevated ARG abundances at certain sites, cannot be excluded. However, additional research applying microbial source tracking and the analysis of antibiotic residues is needed to resolve these uncertainties.

### Exposure and risk assessment

Nevertheless, the presence of ARB and ARG in surface water bears the risk of their transfer to water users, for example by ingestion of water during water-related recreational activities (e. g. [69,70]). Although Lahn River is not officially designated as bathing water, it became very popular for canoeing and boating in recent years [27, 71].

As described earlier, theoretical prevalence of antibiotic-resistant *E*. *coli*, ESBL-producing *E*. *coli* and diarrheagenic ESBL-producing *E*. *coli* was calculated based on prevalence values obtained from the literature [45, 47–48, 54]. Based on these results, amounts of bacteria and ARG potentially ingested during various recreational activities including swimming and non-swimming activities were calculated using water ingestion rates published in literature [28–30]. Reported proportions of antibiotic-resistant *E*. *coli* isolates in several Central European rivers were 32% (Rhine), 34% (New Meuse) and 48% (Meuse) [45]. Antibiotic resistance in *E*. *coli* isolates from the Seine River in France was as high as 42% [72]. *Bla*_CTX-M_ abundance in enteric bacteria in some United Kingdom bathing waters was assumed to be 0.1% [73]. This is within the range reported elsewhere in the literature. Leonard et al. [69] found a prevalence of 3^rd^ generation cephalosporin-resistance in *E*. *coli* in coastal surface waters in England and Wales of 0.12%. In some Dutch recreational waters ESBL-producing *E*. *coli* represented 0.05–1% of the total *E*. *coli* population [46]. Higher values were reported by Haberecht et al. [47], who found 1.7% of *E*. *coli* harboring ESBL resistance in surface water of Cache La Poudre River, USA. 8.5% of all ESBL-producing *E*. *coli* isolates from surface waters in the Netherlands were suspected diarrheagenic variants [48].

Assessment of water sports related exposure revealed that in theory participants in swimming and non-swimming recreational activities incidentally swallow considerable amounts of potentially resistant *E*. *coli* and ARG (Table 3, Table 4; detailed versions of the tables are provided in the supporting information: S1 Table, S2 Table).

**Table 3:** Exposure of water sports participants in Lahn River to (theoretically antibiotic-resistant) *E*. *coli*.

		activity	range	(1) total *E*. *coli*	(2) resistant *E*. *coli*	(3) resistant *E*. *coli*	(4) ESBL *E*. *coli*	(5) ESBL *E*. *coli*	(6) diarrheagenic ESBL *E*. *coli*	(7) diarrheagenic ESBL *E*. *coli*
**prevalence [MPN/100 mL]**			MIN	15	5	7	0	0	0	0
			AVG	3158	1011	1516	2	54	0	5
			MAX	27730	8874	13310	14	471	1	40
**ingested per hour**	**[MPN/h]**	non-swimming^a^	MIN	0	0	0	0	0	0	0
			AVG	125	40	60	0	2	0	0
			MAX	2085	667	1001	1	35	0	3
		swimming (children)^b^	MIN	7	2	4	0	0	0	0
			AVG	1558	499	748	1	26	0	2
			MAX	13679	4377	6566	7	233	1	20
		swimming (adults)^b^	MIN	3	1	2	0	0	0	0
			AVG	674	216	323	0	11	0	1
			MAX	5915	1893	2839	3	101	0	9
**ingested per session**	**[MPN/session]**	non-swimming^c^	MIN	1	0	1	0	0	0	0
			AVG	430	138	206	0	7	0	1
			MAX	5422	1735	2602	3	92	0	8
		swimming (children)^d^	MIN	10	3	5	0	0	0	0
			AVG	2057	658	987	1	35	0	3
			MAX	18057	5778	8667	9	307	1	26
		swimming (adults)^d^	MIN	4	1	2	0	0	0	0
			AVG	889	285	427	0	15	0	1
			MAX	7808	2498	3748	4	133	0	11

(a) based on ingestion rates by Rijal et al. 2011, Dorevitch et al. 2011, Dufour et al. 2011, including boating, canoeing, fishing, kayaking and rowing

(b) based on ingestion rates by Dufour et al. 2011

(c) based on ingestion rates by Rijal et al.2011, Dorevitch et al. 2011, Dufour et al. 2011, durations by Rijal et al. 2011, including boating, canoeing, fishing

(d) based on ingestion rates by Dufour et al. 2011 and average duration by Schets et al. 2011; (1) based on Herrig et al. 2015; (2) based on (1) and Blaak et al. 2011 (32% of *E*. *coli* antibiotic-resistant *E*. *coli*); (3) based on (1) and Blaak et al. 2011 (48% of *E*. *coli* antibiotic-resistant); (4) based on (1) and Blaak et al. 2014 (0.05% of *E*. *coli* producing ESBL); (5) based on (1) and Haberecht et al. 2019 (1.7% of *E*. *coli* producing ESBL); (6) based on (4) and Franz et al. 2015 (8.5% of ESBL producing *E*. *coli* diarrheagenic); (7) based on (5) and Franz et al. 2015 (8.5% of ESBL producing *E*. *coli* diarrheagenic).

**Table 4:** Exposure of water sports participants in Lahn River to *bla*_CTX-M_ genes.

		activity	range	*bla*_CTX-M_ genes
**prevalence [copies/100 mL]**			MIN	349
			AVG	3179
			MAX	20083
**ingested per hour**	**[copies/h]**	non-swimming^a^	MIN	7
			AVG	126
			MAX	1510
		swimming (children)^b^	MIN	172
			AVG	1568
			MAX	9907
		swimming (adults)^b^	MIN	74
			AVG	678
			MAX	4284
**ingested per session**	**[copies/session]**	non-swimming^c^	MIN	27
			AVG	435
			MAX	3927
		swimming (children)^d^	MIN	227
			AVG	2070
			MAX	13077
		swimming (adults)^d^	MIN	98
			AVG	895
			MAX	5654

a) based on ingestion rates by Rijal et al. 2011, Dorevitch et al. 2011, Dufour et al. 2011, including boating, canoeing, fishing, kayaking and rowing

b) based on ingestion rates by Dufour et al. 2011

c) based on ingestion rates by Rijal et al.2011, Dorevitch et al. 2011, Dufour et al. 2011, durations by Rijal et al. 2011, including boating, canoeing, fishing

d) based on ingestion rates by Dufour et al. 2011 and average duration by Schets et al. 2011

Amounts depend on the degree of water contact and the level of water pollution. Unsurprisingly, swimming activities pose a higher risk of exposure as non-swimming water sports, especially for children (Table 3, Table 4). Based on the assumption that 32% to 48% [45] of *E*. *coli* detected in rivers may be antibiotic-resistant, participants are theoretically at risk of ingesting 0–1001 MPN of potentially antibiotic-resistant *E*. *coli* per hour and 0–2602 MPN of potentially antibiotic-resistant *E*. *coli* per session during non-swimming activities (Table 3). Considering that 0.05% [46] to 1.7% [47] of *E*. *coli* in freshwater may carry ESBL genes, 0–35 MPN of potentially ESBL-producing *E*. *coli* may be ingested per hour and 0–92 MPN of potentially ESBL-producing *E*. *coli* may be ingested per non-swimming water sports session (Table 3). Taking into account that 8.5% of all ESBL-producing *E*. *coli* in surface water are suspected to be potential gastrointestinal pathogens (including enteroaggregative and enterotoxigenic *E*. *coli*) [48], participation in swimming can theoretically result in the ingestion of 0–26 MPN of potentially diarrheagenic ESBL-producing *E*. *coli* per children’s swim session (Table 3). However, the probability of infection when ingesting the calculated maximum possible concentration of 26 MPN (Table 3) is actually very small, independently of model parameters used (Table 5).

**Table 5:** Microbial risk assessment for infection with presumptive diarrheagenic ESBL-producing *E*. *coli* for children during swimming in Lahn River at times of peak concentrations.

Reference	Haas et al. 1999	DuPont et al. 1971
Host type	Human	Human
Pathogen type	Non-enterohaemorrhagic strains including ETEC, EPEC, EIEC	EIEC 1624
Response	Diarrhea	Positive stool isolation
Best Fit Model	Beta-Poisson	Beta-Poisson
alpha	1.78E-01	1.55E-01
ND_50_	8.60E+07	2.11E+06
**P**_**(response)**_	**2.59E-06**	**1.65E-04**

Abbreviations: ETEC: enterotoxigenic *E*. *coli*; EPEC: enteropathogenic *E*. *coli*; EIEC: enteroinvasive *E*. *coli*.

Nevertheless, Haas et al. [74] clearly emphasized that even a single microorganism has the potential to cause an infection.

Depending on the degree of water contact and the level of water pollution, up to 1510 copies of *bla*_CTX-M_ genes may be ingested per hour of non-swimming water sports resulting in up to 3927 copies theoretically ingested per session (Table 4). As a worst-case scenario, up to 13,077 copies may be swallowed by children during swimming per session (Table 4).

Yet, results concerning infection risk and exposure to ARB as well as ARG should be interpreted with caution as they are based on several assumptions. Human exposure to calculated maximum numbers might only occur during times of peak pollution. However, these were observed predominantly during fall and winter and shortly after pronounced precipitation events, when less water sports participants can be expected.

Dose-response models used in this study did not focus specifically on antibiotic-resistant strains. Furthermore, the risk assessment conducted included solely theoretically diarrheagenic *E*. *coli* carrying ESBL genes. This likely underestimates the risks posed by total potentially pathogenic ARB present in Lahn River. In relation to *E*. *coli* a high prevalence of *bla*_CTX-M_ genes was measured in Lahn River. In comparison, a relatively low ESBL prevalence in *E*. *coli* is described in the literature [46, 47]. This suggests that the level of resistance conferred by *bla*_CTX-M_ in Lahn River might actually be considerably higher than estimated based on ESBL prevalence in *E*. *coli*. Moreover, transmission of ARG from the environment to humans does not occur solely by pathogens, but in particular by vector bacteria carrying ARG. Risk assessment of the transmission of vector bacteria cannot rely on the same model used for pathogens, because most vectors are non-pathogenic [75]. They may colonize a healthy host without causing disease but infection can break out, if the host’s immune system is compromised [75]. Risk assessment must thus be conducted using vector bacteria instead of their resistance genes [75]. However, there is a lack of knowledge regarding risk assessment in the context of ARB transmission in environmental settings as well as of data quantitatively linking ARG uptake to adverse health outcomes [76]. Although this study cannot relate the concentrations of *bla*_CTX-M_ genes measured to any infection risk, the presence of ARG and ARB is associated with the risk of being transferred to the human bacterial flora, even by nonpathogenic harmless bacteria. *E*. *coli* can act as a vector transferring ARG between environment and host as well as *in vivo* [70, 77]. Swallowing water contaminated with *bla*_CTX-M_-bearing *E*. *coli* was shown to be linked to gut colonization by these bacteria in surfers [70]. In addition, transfer of plasmids carrying *bla*_CTX-M_ between *E*. *coli* within the human gut has been described [77]. As participants in water related recreation at Lahn River are at risk of swallowing considerable amounts of *bla*_CTX-M_ genes and *E*. *coli*, they might become colonized by ARB. To further validate the assumptions made on prevalence and ingestion rates in this study, data on proportions of *bla*_CTX-M_ carrying *E*. *coli* and antibiotic-resistant *E*. *coli* should be obtained directly from Lahn River water.

### *E*. *coli* as estimator for ARG concentrations

**Fig 6:**
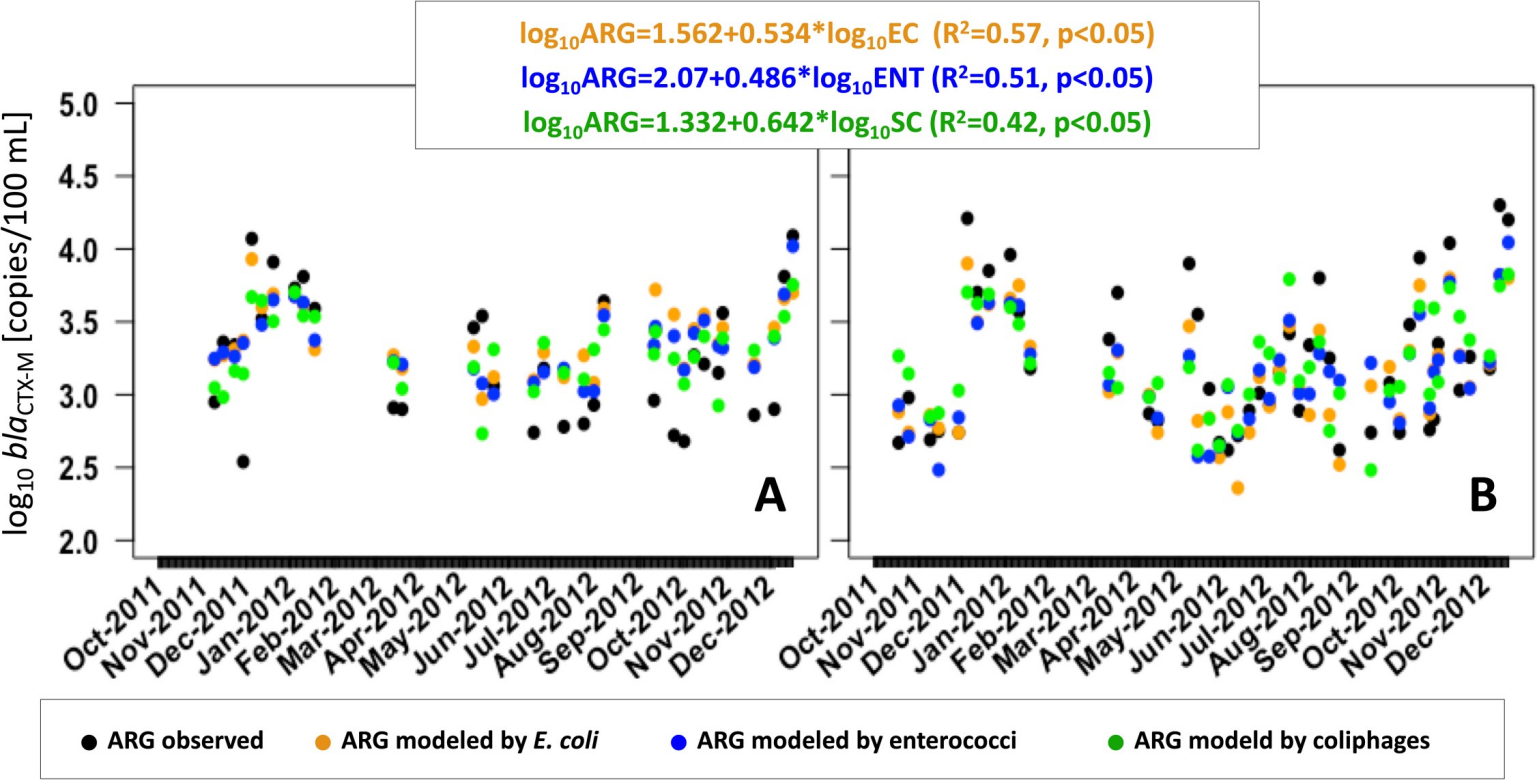
Linear model equations and measured and modeled *bla*_CTX-M_ ARG concentrations during the sampling period at (A) site 1 and (B) site 2.

**Fig 7:**
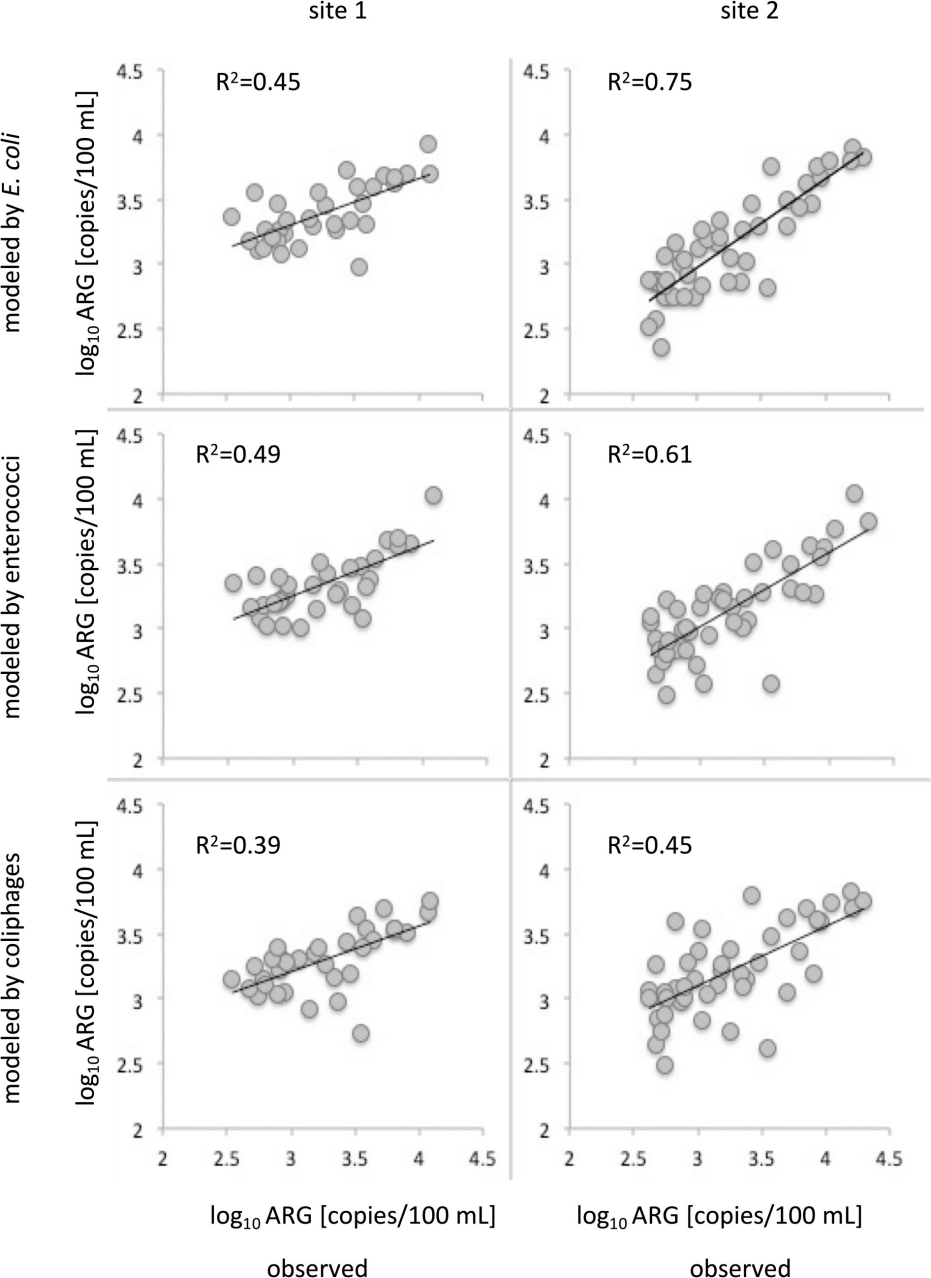
Modeled vs. observed concentrations of *bla*_CTX-M_ ARG for each individual sampling site.

Even if the risk of acquiring gastrointestinal infections due to ESBL-carrying diarrheagenic *E*. *coli* is very low, results show that transmission of ARG and theoretically also antibiotic-resistant *E*. *coli* to humans via contact with river water during recreational activities is a realistic scenario. To prevent exposure or to identify times of enhanced risks of human exposure, predictive models allowing a timely assessment of microbial pollution with ARB or ARG would be beneficial. It was suggested that FIB might serve as a suitable estimate for the presence of ARB strains [23, 52] as FIB are commonly used in water quality assessment. In accordance with other studies (e.g. [78]), the presence of ARG could largely be explained by fecal pollution (Table 2). Hence, linear regression models based on FIO were able to explain *bla*_CTX-M_ concentrations with varying accuracy depending on type of FIO and study site (Fig 6, Fig 7).

Notably, ARG concentrations can be estimated by FIO with a considerably higher accuracy at site 2 (Fig 7). As expected, *E*. *coli* provided the best estimates for ARG in Lahn River, with three-quarters (75%) of variance explained. Yet, further model validation using an independent reference dataset will be required. ARG and FIO were shown to be similarly distributed and were influenced by environmental factors in a similar way. Thus, multiple linear regression models (MLR) as previously established for the prediction of FIO based on environmental factors [26, 54, 79] may also be suitable for the prediction of ARG. To close the knowledge gap between the prevalence of *bla*_CTX-M_ genes and the actual level of antibiotic resistance, a variety of bacterial isolates should be tested for antibiotic resistances in future studies and correlations between *bla*_CTX-M_ genes should be analyzed. Results could strengthen the assumptions made on the suitability of *bla*_CTX-M_ genes as proxy for the total level of antibiotic resistance in river environments.

## Conclusions

*Bla*_CTX-M_ genes were found to be omnipresent in Lahn River surface water. Overall, *E*. *coli* and *bla*_CTX-M_ genes followed a similar trend and their abundances varied according to temporal variations in hydro-meteorological factors. An influence of WWTP discharges on *bla*_CTX-M_ gene levels was observed under low flow conditions, whereas total concentrations of *bla*_CTX-M_ genes increased after rainfall events in accordance with the degree of agricultural impact in the surrounding catchment. In general, participants in swimming and non-swimming recreational activities (i.e. boating, fishing, canoeing) at Lahn River are at risk of ingesting ARB and ARG. Despite the limitation that the amount of ARG and FIO ingested cannot be linked to an actual risk of infection, results demonstrate potential health risks associated with microbial water quality and water related recreational activities. *Bla*_CTX-M_ gene abundance was largely explained by fecal pollution, with *E*. *coli* providing the best estimates. This information will be helpful in the fields of risk assessment and water management.

## Supporting information

S1 Table Exposure of water sports participants in Lahn River to (theoretically antibiotic resistant) *E*. *coli*. (PDF)

S2 Table Exposure of water sports participants in Lahn River to *bla*_CTX-M_ antibiotic resistance genes. (PDF)

## References

[1] Cassini A, Högberg LD, Plachouras D, Quattrocchi A, Hoxha A, Simonsen GS, et al Attributable deaths and disability-adjusted life-years caused by infections with antibiotic-resistant bacteria in the EU and the European Economic Area in 2015: a population-level modelling analysis. Lancet Infect Dis. 2019 1;19(1):56–66. 10.1016/S1473-3099(18)30605-4 30409683 PMC6300481

[2] O’Neill J. Tackling drug-resistant infections globally: Final report and recommendations [Internet]. HM Government; 2016 [cited 2019 Oct 17]. Available from: https://amr-review.org

[3] Bassetti M, Nicolini L, Esposito S, Righi E, Viscoli C. Current status of newer carbapenems. Curr Med Chem. 2009 2 1;16(5):564–75. 10.2174/092986709787458498 19199922

[4] Hawkey PM, Jones AM. The changing epidemiology of resistance. J Antimicrob Chemother. 2009 9 1;64(Supplement 1):i3–10. 10.1093/jac/dkp256 19675017

[5] McKenna M. Antibiotic resistance: the last resort. Nat News. 2013;499(7459):394–396. 10.1038/499394a 23887414

[6] Paterson DL. Resistance in gram-negative bacteria: *Enterobacteriaceae*. Am J Infect Control. 2006;34(5):S20–8. 10.1016/j.amjmed.2006.03.013 16813978

[7] Pfeifer Y, Eller C, Leistner R, Valenza G, Nickel S, Guerra B, et al ESBL-Bildner als Infektionserreger beim Menschen und die Frage nach dem zoonotischen Reservoir. Hyg Med. 2013:38(7/8):294–99. German. 10.25646/1735

[8] Bevan ER, Jones AM, Hawkey PM. Global epidemiology of CTX-M β-lactamases: temporal and geographical shifts in genotype. J Antimicrob Chemother. 2017 5 25;72(8):2145–55. 10.1093/jac/dkx146 28541467

[9] Livermore DM, Canton R, Gniadkowski M, Nordmann P, Rossolini GM, Arlet G, et al CTX-M: changing the face of ESBLs in Europe. J Antimicrob Chemother. 2006 12 6;59(2):165–74. 10.1093/jac/dkl483 17158117

[10] Carattoli A. Resistance plasmid families in *Enterobacteriaceae*. Antimicrob Agents Chemother. 2009 6 1;53(6):2227 10.1128/AAC.01707-08 19307361 PMC2687249

[11] Robin F, Beyrouthy R, Bonacorsi S, Aissa N, Bret L, Brieu N, et al Inventory of Extended-Spectrum-β-Lactamase-Producing *Enterobacteriaceae* in France as Assessed by a Multicenter Study. Antimicrob Agents Chemother. 2017 2 23;61(3):e01911–16. 10.1128/AAC.01911-16 27956424 PMC5328551

[12] Blaak H, Lynch G, Italiaander R, Hamidjaja RA, Schets FM, de Roda Husman AM. Multidrug-resistant and extended spectrum beta-lactamase-producing *Escherichia coli* in Dutch surface water and wastewater. Mokrousov I, editor. PLOS ONE. 2015 6 1;10(6):e0127752 10.1371/journal.pone.0127752 26030904 PMC4452230

[13] Jørgensen SB, Søraas AV, Arnesen LS, Leegaard TM, Sundsfjord A, Jenum PA. A comparison of extended spectrum β-lactamase producing *Escherichia coli* from clinical, recreational water and wastewater samples associated in time and location. PLOS ONE. 2017 10 17;12(10):e0186576 10.1371/journal.pone.0186576 29040337 PMC5645111

[14] Zhang X-X, Zhang T, Fang HHP. Antibiotic resistance genes in water environment. Appl Microbiol Biotechnol. 2009 3 1;82(3):397–414. 10.1007/s00253-008-1829-z 19130050

[15] Zurfluh K, Abgottspon H, Hächler H, Nüesch-Inderbinen M, Stephan R. Quinolone Resistance Mechanisms among Extended-Spectrum Beta-Lactamase (ESBL) Producing *Escherichia coli* Isolated from Rivers and Lakes in Switzerland. PLoS ONE. 2014 4 22;9(4):e95864 10.1371/journal.pone.0095864 24755830 PMC3995870

[16] Baquero F, Martínez J-L, Cantón R. Antibiotics and antibiotic resistance in water environments. Energy Biotechnol Environ Biotechnol. 2008 6 1;19(3):260–5. 10.1016/j.copbio.2008.05.006 18534838

[17] Okeke IN, Edelman R. Dissemination of Antibiotic-Resistant Bacteria across Geographic Borders. Clin Infect Dis. 2001 8 1;33(3):364–9. 10.1086/321877 11438903

[18] Rath S, Patra B. Dispersal of Antibiotic Resistant Bacteria into Aquatic Environment—An Overview. J Water Pollut Control. 2018 1 1:1(2):1–3.

[19] Iversen A, Kühn I, Rahman M, Franklin A, Burman LG, Olsson-Liljequist B, et al Evidence for transmission between humans and the environment of a nosocomial strain of *Enterococcus faecium*. Environ Microbiol. 2004 1 1;6(1):55–9. 10.1046/j.1462-2920.2003.00534.x 14686941

[20] Laurens C, Jean-Pierre H, Licznar-Fajardo P, Hantova S, Godreuil S, Martinez O, et al Transmission of IMI-2 carbapenemase-producing *Enterobacteriaceae* from river water to human. J Glob Antimicrob Resist. 2018 12 1;15:88–92. 10.1016/j.jgar.2018.06.022 30279153

[21] Baquero F, Alvarez-Ortega C, Martinez JL. Ecology and evolution of antibiotic resistance. Environ Microbiol Rep. 2009 12 1;1(6):469–76. 10.1111/j.1758-2229.2009.00053.x 23765924

[22] von Wintersdorff CJH, Penders J, van Niekerk JM, Mills ND, Majumder S, van Alphen LB, et al Dissemination of Antimicrobial Resistance in Microbial Ecosystems through Horizontal Gene Transfer. Front Microbiol. 2016 2 19;7:173–173. 10.3389/fmicb.2016.00173 26925045 PMC4759269

[23] Berendonk TU, Manaia CM, Merlin C, Fatta-Kassinos D, Cytryn E, Walsh F, et al Tackling antibiotic resistance: the environmental framework. Nat Rev Microbiol. 2015 5;13(5):310–7. 10.1038/nrmicro3439 25817583

[24] Larsson DGJ, Andremont A, Bengtsson-Palme J, Brandt KK, de Roda Husman AM, Fagerstedt P, et al Critical knowledge gaps and research needs related to the environmental dimensions of antibiotic resistance. Environ Int. 2018 8 1;117:132–8. 10.1016/j.envint.2018.04.041 29747082

[25] Tacão M, Correia A, Henriques I. Resistance to Broad-Spectrum Antibiotics in Aquatic Systems: Anthropogenic Activities Modulate the Dissemination of *bla* _CTX-M_ -Like Genes. Appl Environ Microbiol. 2012 6 15;78(12):4134–40. 10.1128/AEM.00359-12 22492443 PMC3370516

[26] Herrig IM, Böer SI, Brennholt N, Manz W. Development of multiple linear regression models as predictive tools for fecal indicator concentrations in a stretch of the lower Lahn River, Germany. Water Res. 2015 11;85:148–57. 10.1016/j.watres.2015.08.006 26318647

[27] BMWi. Die wirtschaftlichen Potenziale des Wassertourismus in Deutschland. Berlin, Germany: Bundesministerium für Wirtschaft und Energie; 2016 12 German. Available from: https://www.bmwi.de/Redaktion/DE/Publikationen/Tourismus/potenziale-des-wassertourismus-in-deutschland.pdf?__blob=publicationFile&v=12

[28] Dorevitch S, Panthi S, Huang Y, Li H, Michalek AM, Pratap P, et al Water ingestion during water recreation. Water Res. 2011 2 1;45(5):2020–8. 10.1016/j.watres.2010.12.006 21227479

[29] Dufour AP, Evans O, Behymer TD, Cantu R. Water ingestion during swimming activities in a pool: a pilot study. J Water Health. 2006 12;4(4):425–30. 10.2166/wh.2006.0026 17176813

[30] Rijal G, Tolson JK, Petropoulou C, Granato TC, Glymph A, Gerba C, et al Microbial risk assessment for recreational use of the Chicago Area Waterway System. J Water Health. 2011 3;9(1):169–86. 10.2166/wh.2010.020 21301125

[31] BfG. Gewässerkundliches Jahrbuch Rheingebiet, Teil III, Abflüsse 2011 [Internet]. 2011 [cited 2019 Dec 11]. Available from: http://dgj-daten.bafg.de/Rhein/Kalkofen/25800600_WQ.pdf

[32] Statistisches Bundesamt. Online-Flächenatlas [Internet]. 2020 [cited 2020 Apr 2]. Available from: https://www.destatis.de/DE/Service/Statistik-Visualisiert/flaechenatlas.html

[33] Drewes JE, Karakurt S, Schmid L, Bachmaier M, Hübner U, Clausnitzer V, et al Dynamik der Klarwasseranteile in Oberflächengewässern und mögliche Herausforderung für die Trinkwassergewinnung in Deutschland. Dessau-Roßlau, Germany: Umweltbundesamt; 2018 7 No. 59/2018. German. Available from: http://www.umweltbundesamt.de/publikationen

[34] MUEEF [Internet]. Geoportal Wasser RLP [cited 2019 Sep 4]. Available from: https://geoportal-wasser.rlp-umwelt.de/servlet/is/2025/

[35] Li A-D, Metch JW, Wang Y, Garner E, Zhang AN, Riquelme MV, et al Effects of sample preservation and DNA extraction on enumeration of antibiotic resistance genes in wastewater. FEMS Microbiol Ecol. 2018 2 1;94(2). 10.1093/femsec/fix189 29300934

[36] Marti E, Jofre J, Balcazar JL. Prevalence of Antibiotic Resistance Genes and Bacterial Community Composition in a River Influenced by a Wastewater Treatment Plant. PLOS ONE. 2013 10 25;8(10):e78906 10.1371/journal.pone.0078906 24205347 PMC3808343

[37] Kim J, Lim Y-M, Jeong Y-S, Seol S-Y. Occurrence of CTX-M-3, CTX-M-15, CTX-M-14, and CTX-M-9 extended-spectrum beta-lactamases in *Enterobacteriaceae* clinical isolates in Korea. Antimicrob Agents Chemother. 2005 4;49(4):1572–5. 10.1128/AAC.49.4.1572-1575.2005 15793142 PMC1068616

[38] Büchter B. Vorkommen und Charakterisierung von Extended-Spectrum-Beta-Laktamase (ESBL)-produzierenden *Escherichia coli* bei Lebensmittel liefernden Tieren [dissertation]. Freie Universität Berlin; 2011 10.17169/refubium-5158

[39] Coque TM, Oliver A, Pérez-Díaz JC, Baquero F, Cantón R. Genes encoding TEM-4, SHV-2, and CTX-M-10 extended-spectrum beta-lactamases are carried by multiple *Klebsiella pneumoniae* clones in a single hospital (Madrid, 1989 to 2000). Antimicrob Agents Chemother. 2002 2;46(2):500–10. 10.1128/AAC.46.2.500-510.2002 11796363 PMC127031

[40] NCBI [Internet]. *Escherichia coli*: A well-studied enteric bacterium. 2019 [cited 2019 Feb 19]. Available from: https://www.ncbi.nlm.nih.gov/genome/?term=escherichia%20coli

[41] ISO. ISO 9308–3: Water quality—Detection and enumeration of *Escherichia coli* and coliform bacteria in surface and waste water–Part 3: Miniaturized method (most probable number) by inoculation in liquid medium. Geneva, Switzerland: International Organization for Standardization; 1998 10.31030/8106465

[42] ISO. ISO 7899–2: Water quality—Detection and enumeration of intestinal enterococci—Part 2: Membrane filtration method. Geneva, Switzerland: International Organization for Standardization; 2000 10.31030/8974114

[43] ISO. ISO 10705–2: Water quality—Detection and enumeration of bacteriophages—Part 2: Enumeration of somatic coliphages. Geneva, Switzerland: International Organization for Standardization; 2000 10.31030/9224349

[44] R Core Team. R: a language and environment for statistical computing. Version 3.5.1 [software]. Vienna, Austria: Foundation for Statistical Computing; 2018 Available from: https://www.R-project.org/

[45] Blaak, van Rooijen SR, Schuijt MS, van Leeuwen AE, Italiaander R, van den Berg HJL, et al. Prevalence of antibiotic resistant bacteria in the rivers Meuse, Rhine and New Meuse. RIVM Report 703719071/2011. Bilthoven, Netherlands: National Insitute for Public Health and the Environment; 2011. Available from: https://www.rivm.nl/bibliotheek/rapporten/703719071.html

[46] Blaak H, de Kruijf P, Hamidjaja RA, van Hoek AHAM, de Roda Husman AM, Schets FM. Prevalence and characteristics of ESBL-producing *E*. *coli* in Dutch recreational waters influenced by wastewater treatment plants. Vet Microbiol. 2014 7 16;171(3):448–59. 10.1016/j.vetmic.2014.03.007 24690376

[47] Haberecht HB, Nealon NJ, Gilliland JR, Holder AV, Runyan C, Oppel RC, et al Antimicrobial-Resistant *Escherichia coli* from Environmental Waters in Northern Colorado. J Environ Public Health. 2019 2 18;2019:1–13. 10.1155/2019/3862949 30906330 PMC6397973

[48] Franz E, Veenman C, van Hoek AHAM, Husman A de R, Blaak H. Pathogenic *Escherichia coli* producing Extended-Spectrum β-Lactamases isolated from surface water and wastewater. Sci Rep. 2015 9 24;5:14372 10.1038/srep14372 26399418 PMC4585870

[49] Schets FM, Schijven JF, de Roda Husman AM. Exposure assessment for swimmers in bathing waters and swimming pools. Water Res. 2011 3 1;45(7):2392–400. 10.1016/j.watres.2011.01.025 21371734

[50] Haas CN, Rose JB, Gerba CP. Quantitative microbial risk assessment. New York: John Wiley & Sons; 1999.

[51] DuPont HL, Formal SB, Hornick RB, Snyder MJ, Libonati JP, Sheahan DG, et al Pathogenesis of *Escherichia coli* diarrhea. N Engl J Med. 1971 7 1;285(1):1–9. 10.1056/NEJM197107012850101 4996788

[52] Gekenidis M-T, Qi W, Hummerjohann J, Zbinden R, Walsh F, Drissner D. Antibiotic-resistant indicator bacteria in irrigation water: High prevalence of extended-spectrum beta-lactamase (ESBL)-producing *Escherichia coli*. PLOS ONE. 2018 11 26;13(11):e0207857 10.1371/journal.pone.0207857 30475879 PMC6258136

[53] McConnell MM, Hansen LT, Neudorf KD, Hayward JL, Jamieson RC, Yost CK, et al Sources of Antibiotic Resistance Genes in a Rural River System. J Environ Qual. 2018;47(5):997 10.2134/jeq2017.12.0477 30272774

[54] Herrig I, Seis W, Fischer H, Regnery J, Manz W, Reifferscheid G, et al Prediction of fecal indicator organism concentrations in rivers: the shifting role of environmental factors under varying flow conditions. Environ Sci Eur. 2019 9 23;31(1):59 10.1186/s12302-019-0250-9

[55] Gothwal R, Shashidhar T. Antibiotic Pollution in the Environment: A Review. CLEAN–Soil Air Water. 2015 4 1;43(4):479–89. 10.1002/clen.201300989

[56] Sabri NA, Schmitt H, Van der Zaan B, Gerritsen HW, Zuidema T, Rijnaarts HHM, et al Prevalence of antibiotics and antibiotic resistance genes in a wastewater effluent-receiving river in the Netherlands. J Environ Chem Eng. [preprint] 2018 3 12 10.1016/j.jece.2018.03.004

[57] Khan FA, Söderquist B, Jass J. Prevalence and Diversity of Antibiotic Resistance Genes in Swedish Aquatic Environments Impacted by Household and Hospital Wastewater. Front Microbiol. 2019;10:688 10.3389/fmicb.2019.00688 31019498 PMC6458280

[58] Amos GCA, Zhang L, Hawkey PM, Gaze WH, Wellington EM. Functional metagenomic analysis reveals rivers are a reservoir for diverse antibiotic resistance genes. Vet Microbiol. 2014 7;171(3–4):441–7. 10.1016/j.vetmic.2014.02.017 24636906

[59] Knapp CW, Lima L, Olivares-Rieumont S, Bowen E, Werner D, Graham DW. Seasonal Variations in Antibiotic Resistance Gene Transport in the Almendares River, Havana, Cuba. Front Microbiol. 2012 11; 3:396 10.3389/fmicb.2012.00396 23189074 PMC3505016

[60] Beattie RE, Walsh M, Cruz MC, McAliley LR, Dodgen L, Zheng W, et al Agricultural contamination impacts antibiotic resistance gene abundances in river bed sediment temporally. FEMS Microbiol Ecol. 2018 7 13;94(9):fiy131 10.1093/femsec/fiy131 30010841

[61] Chen B, Liang X, Huang X, Zhang T, Li X. Differentiating anthropogenic impacts on ARGs in the Pearl River Estuary by using suitable gene indicators. Water Res. 2013 5;47(8):2811–20. 10.1016/j.watres.2013.02.042 23521975

[62] Pruden A, Pei R, Storteboom H, Carlson KH. Antibiotic Resistance Genes as Emerging Contaminants: Studies in Northern Colorado. Environ Sci Technol. 2006 12 1;40(23):7445–50. 10.1021/es060413l 17181002

[63] Pietramellara G, Ascher J, Borgogni F, Ceccherini MT, Guerri G, Nannipieri P. Extracellular DNA in soil and sediment: fate and ecological relevance. Biol Fertil Soils. 2009 2 1;45(3):219–35. 10.1007/s00374-008-0345-8

[64] Mao D, Luo Y, Mathieu J, Wang Q, Feng L, Mu Q, et al Persistence of Extracellular DNA in River Sediment Facilitates Antibiotic Resistance Gene Propagation. Environ Sci Technol. 2014 1 7;48(1):71–8. 10.1021/es404280v 24328397

[65] Nielsen KM, Johnsen PJ, Bensasson D, Daffonchio D. Release and persistence of extracellular DNA in the environment. Environ Biosafety Res. 2007;6(1–2):37–53. 10.1051/ebr:2007031 17961479

[66] Agnelli A, Ascher J, Corti G, Ceccherini MT, Nannipieri P, Pietramellara G. Distribution of microbial communities in a forest soil profile investigated by microbial biomass, soil respiration and DGGE of total and extracellular DNA. Soil Biol Biochem. 2004 5 1;36(5):859–68. 10.1016/j.soilbio.2004.02.004

[67] Ceccherini MT, Ascher J, Pietramellara G, Vogel TM, Nannipieri P. Vertical advection of extracellular DNA by water capillarity in soil columns. Soil Biol Biochem. 2007 1 1;39(1):158–63. 10.1016/j.soilbio.2006.07.006

[68] Poté J, Ceccherini MT, Van VT, Rosselli W, Wildi W, Simonet P, et al Fate and transport of antibiotic resistance genes in saturated soil columns. Eur J Soil Biol. 2003 4 1;39(2):65–71. 10.1016/S1164-5563(03)00003-7

[69] Leonard AFC, Zhang L, Balfour AJ, Garside R, Gaze WH. Human recreational exposure to antibiotic resistant bacteria in coastal bathing waters. Environ Int. 2015 9;82:92–100. 10.1016/j.envint.2015.02.013 25832996

[70] Leonard AFC, Zhang L, Balfour AJ, Garside R, Hawkey PM, Murray AK, et al Exposure to and colonisation by antibiotic-resistant *E*. *coli* in UK coastal water users: Environmental surveillance, exposure assessment, and epidemiological study (Beach Bum Survey). Environ Int. 2018 5;114:326–33. 10.1016/j.envint.2017.11.003 29343413

[71] Biedenkapp A, Stührmann E. Tourismus, Naturschutz und Wassersport: Dokumentation der Fachtagung am 7 Februar 2004 im Rahmen des 14. Reisepavillon, Hannover. BfN-Skripten 113. Bonn, Germany: Bundesamt für Naturschutz; 2004 German. Available from: https://www.bfn.de/infothek/veroeffentlichungen/bfn-skripten/sport-tourismus.html

[72] Servais P, Passerat J. Antimicrobial resistance of fecal bacteria in waters of the Seine river watershed (France). Sci Total Environ. 2009 12;408(2):365–72. 10.1016/j.scitotenv.2009.09.042 19853889

[73] Finley RL, Collignon P, Larsson DGJ, McEwen SA, Li X-Z, Gaze WH, et al The Scourge of Antibiotic Resistance: The Important Role of the Environment. Clin Infect Dis. 2013 5 30;57(5):704–10. 10.1093/cid/cit355 23723195

[74] Haas CN. Estimation of risk due to low doses of microorganisms: a comparison of alternative methodologies. Am J Epidemiol. 1983 Oktober;118(4):573–82. 10.1093/oxfordjournals.aje.a113662 6637984

[75] Manaia CM. Assessing the Risk of Antibiotic Resistance Transmission from the Environment to Humans: Non-Direct Proportionality between Abundance and Risk. Trends Microbiol. 2017 März;25(3):173–81. 10.1016/j.tim.2016.11.014 28012687

[76] Ashbolt NJ, Amézquita A, Backhaus T, Borriello P, Brandt KK, Collignon P, et al Human Health Risk Assessment (HHRA) for environmental development and transfer of antibiotic resistance. Environ Health Perspect. 2013 9;121(9):993–1001. 10.1289/ehp.1206316 23838256 PMC3764079

[77] Knudsen PK, Gammelsrud KW, Alfsnes K, Steinbakk M, Abrahamsen TG, Müller F, et al Transfer of a bla(CTX-M-1)-carrying plasmid between different Escherichia coli strains within the human gut explored by whole genome sequencing analyses. Sci Rep. 2018 1 10;8(1):280–280. 10.1038/s41598-017-18659-2 29321570 PMC5762863

[78] Karkman A, Pärnänen K, Larsson DGJ. Fecal pollution can explain antibiotic resistance gene abundances in anthropogenically impacted environments. Nat Commun. 2019 1 8;10(1):80 10.1038/s41467-018-07992-3 30622259 PMC6325112

[79] Seis W, Zamzow M, Caradot N, Rouault P. On the implementation of reliable early warning systems at European bathing waters using multivariate Bayesian regression modelling. Water Res. 2018 10;143:301–12. 10.1016/j.watres.2018.06.057 29986240

