# Supplementary material for: Prevalence and seasonal dynamics of blaCTX-M antibiotic resistance genes and fecal indicator organisms in the lower Lahn River, Germany
Source: PLoS One. 2020 Apr 30;15(4):e0232289. doi: 10.1371/journal.pone.0232289 (PMC7192499; doi:10.1371/journal.pone.0232289)
Supplement: S1 Table — (PDF) [file pone.0232289.s001.pdf]

Supporting Information

Prevalence and seasonal dynamics of *bla*$_{CTX-M}$ antibiotic resistance genes and fecal indicator organisms in the lower Lahn River, Germany

Ilona Herrig[1,2*], Susanne Fleischmann[1, #a] , Julia Regnery[1], Jessica Wesp[1], Georg Reifferscheid[1], Werner Manz[2]

[1] Department Biochemistry, Ecotoxicology, Federal Institute of Hydrology, Koblenz, Germany

[2] Department of Biology, Institute for Integrated Natural Sciences, University of Koblenz-Landau, Koblenz, Germany

[#a] Current Address: Department of Veterinary Medicine, Institute of Food Safety and Food Hygiene, FU Berlin, Germany

* Corresponding author

E-mail: iherrig@uni-koblenz.de (IH)

S1 Table: Exposure of water sports participants in Lahn River to (antibiotic resistant) *E. coli*.

				prevalence							ingested per hour							ingested per session						
Activity	Volume of water ingested [mL/h]	Duration session [h]	Range	(1) total *E. coli* [MPN/100 mL]	(2) resistant *E. coli* [MPN/100 mL]	(3) resistant E. coli [MPN/100 mL]	(4) ESBL *E. coli* [MPN/100 mL]	(5) ESBL *E. coli* [MPN/100 mL]	(6) diarrheagenic ESBL *E. coli* [MPN/100 mL]	(7) diarrheagenic ESBL *E. coli* [MPN/100 mL]	(1) total *E. coli* [MPN/h]	(2) resistant *E. coli* [MPN/h]	(3) resistant E. coli [MPN/h]	(4) ESBL *E. coli* [MPN/h]	(5) ESBL *E. coli* [MPN/h]	(6) diarrheagenic ESBL *E. coli* [MPN/h]	(7) diarrheagenic ESBL *E. coli* [MPN/h]	(1) total *E. coli* [MPN/session]	(2) resistant E. coli [MPN/session]	(3) resistant E. coli [MPN/session]	(4) ESBL *E. coli* [MPN/session]	(5) ESBL *E. coli* [MPN/session]	(6) diarrheagenic ESBL *E. coli* [MPN/session]	(7) diarrheagenic ESBL *E. coli* [MPN/session]
Boating	3.7[a]	4[b]	min	15	5	7	0	0	0	0	1	0	0	0	0	0	0	2	1	1	0	0	0	0
			avg	3158	1011	1516	2	54	0	5	117	37	56	0	2	0	0	467	150	224	0	8	0	1
			max	27730	8874	13310	14	471	1	40	1026	328	492	1	17	0	1	4104	1313	1970	2	70	0	6
Canoeing	3.9[a]	2.6[b]	min	15	5	7	0	0	0	0	1	0	0	0	0	0	0	2	0	1	0	0	0	0
			avg	3158	1011	1516	2	54	0	5	123	39	59	0	2	0	0	320	102	154	0	5	0	0
			max	27730	8874	13310	14	471	1	40	1081	346	519	1	18	0	2	2812	900	1350	1	48	0	4
Fishing	3.6[a]	4[b]	min	15	5	7	0	0	0	0	1	0	0	0	0	0	0	2	1	1	0	0	0	0
			avg	3158	1011	1516	2	54	0	5	114	36	55	0	2	0	0	455	146	218	0	8	0	1
			max	27730	8874	13310	14	471	1	40	998	319	479	0	17	0	1	3993	1278	1917	2	68	0	6
Kayaking	3.8[a]	-	min	15	5	7	0	0	0	0	1	0	0	0	0	0	0	-	-	-	-	-	-	-
			avg	3158	1011	1516	2	54	0	5	120	38	58	0	2	0	0	-	-	-	-	-	-	-
			max	27730	8874	13310	14	471	1	40	1054	337	506	1	18	0	2	-	-	-	-	-	-	-
Rowing	3.5[a]	-	min	15	5	7	0	0	0	0	1	0	0	0	0	0	0	-	-	-	-	-	-	-
			avg	3158	1011	1516	2	54	0	5	111	35	53	0	2	0	0	-	-	-	-	-	-	-
			max	27730	8874	13310	14	471	1	40	971	311	466	0	16	0	1	-	-	-	-	-	-	-
Boating	1.9[b]	4[b]	min	15	5	7	0	0	0	0	0	0	0	0	0	0	0	1	0	1	0	0	0	0
			avg	3158	1011	1516	2	54	0	5	60	19	29	0	1	0	0	240	77	115	0	4	0	0
			max	27730	8874	13310	14	471	1	40	527	169	253	0	9	0	1	2107	674	1012	1	36	0	3
Fishing	3.79[b]	4[b]	min	15	5	7	0	0	0	0	1	0	0	0	0	0	0	2	1	1	0	0	0	0
			avg	3158	1011	1516	2	54	0	5	120	38	57	0	2	0	0	479	153	230	0	8	0	1
			max	27730	8874	13310	14	471	1	40	1051	336	504	1	18	0	2	4204	1345	2018	2	71	0	6
Canoeing	7.52[b]	2.6[b]	min	15	5	7	0	0	0	0	1	0	1	0	0	0	0	3	1	1	0	0	0	0
			avg	3158	1011	1516	2	54	0	5	237	76	114	0	4	0	0	617	198	296	0	10	0	1
			max	27730	8874	13310	14	471	1	40	2085	667	1001	1	35	0	3	5422	1735	2602	3	92	0	8
Swimming (children)	49.33[c]	1.32[d]	min	15	5	7	0	0	0	0	7	2	4	0	0	0	0	10	3	5	0	0	0	0
			avg	3158	1011	1516	2	54	0	5	1558	499	748	1	26	0	2	2057	658	987	1	35	0	3
			max	27730	8874	13310	14	471	1	40	13679	4377	6566	7	233	1	20	18057	5778	8667	9	307	1	26
Swimming (adults)	21.33[c]	1.32[d]	min	15	5	7	0	0	0	0	3	1	2	0	0	0	0	4	1	2	0	0	0	0
			avg	3158	1011	1516	2	54	0	5	674	216	323	0	11	0	1	889	285	427	0	15	0	1
			max	27730	8874	13310	14	471	1	40	5915	1893	2839	3	101	0	9	7808	2498	3748	4	133	0	11

(1) Herrig et al. 2015
(2) calculated based on (1) and Blaak et al. 2011 (32 % of *E. coli* AB-resistant *E. coli*)
(3) calculated based on (1) and Blaak et al. 2011 (48 % of *E. coli* AB-resistant)
(4) calculated based on (1) and Blaak et al. 2014 (0.05 % of *E. coli* producing ESBL)
(5) calculated based on (1) and Haberecht et al. 2019 (1.7 % of *E. coli* producing ESBL)
(6) calculated based on (4) and Franz et al. 2015 (8.5 % of ESBL producing *E. coli* diarrheagenic)
(7) calculated based on (5) and Franz et al. 2015 (8.5 % of ESBL producing *E. coli* diarrheagenic)

a) Dorevitch et al. 2011
b) Rijal et al. 2011
c) Dufour et al. 2006
d) Schets et al. 2011
