# Supplementary material for: Prevalence and seasonal dynamics of blaCTX-M antibiotic resistance genes and fecal indicator organisms in the lower Lahn River, Germany
Source: PLoS One. 2020 Apr 30;15(4):e0232289. doi: 10.1371/journal.pone.0232289 (PMC7192499; doi:10.1371/journal.pone.0232289)
Supplement: S2 Table — (PDF) [file pone.0232289.s002.pdf]

Supporting Information

Prevalence and seasonal dynamics of *bla*$_{CTX-M}$ antibiotic resistance genes and fecal indicator organisms in the lower Lahn River, Germany

Ilona Herrig[1,2*], Susanne Fleischmann[1, #a], Julia Regnery[1], Jessica Wesp[1], Georg Reifferscheid[1], Werner Manz[2]

[1] Department Biochemistry, Ecotoxicology, Federal Institute of Hydrology, Koblenz, Germany

[2] Department of Biology, Institute for Integrated Natural Sciences, University of Koblenz-Landau, Koblenz, Germany

[#a] Current Address: Department of Veterinary Medicine, Institute of Food Safety and Food Hygiene, FU Berlin, Germany

* Corresponding author

E-mail: iherrig@uni-koblenz.de (IH)

S2 Table: Exposure of water sports participants in Lahn River to *bla*$_{CTX-M}$ antibiotic resistance genes.

				prevalence	ingested per hour	ingested per session
Activity	Volume of water ingested [mL/h]	Duration session [h]	Range	*bla*$_{CTX-M}$ [copies/100 mL]	*bla*$_{CTX-M}$ [copies/h]	*bla*$_{CTX-M}$ [copies/session]
Boating	3.7[a]	4[b]	min	349	13	52
			avg	3179	118	470
			max	20083	743	2972
Canoeing	3.9[a]	2.6[b]	min	349	14	35
			avg	3179	124	335
			max	20083	783	2115
Fishing	3.6[a]	4[b]	min	349	13	50
			avg	3179	114	458
			max	20083	723	2892
Kayaking	3.8[a]	-	min	349	13	-
			avg	3179	121	-
			max	20083	763	-
Rowing	3.5[a]	-	min	349	12	-
			avg	3179	111	-
			max	20083	703	-
Boating	1.9[b]	4[b]	min	349	7	27
			avg	3179	60	242
			max	20083	382	1526
Fishing	3.79[b]	4[b]	min	349	13	53
			avg	3179	120	482
			max	20083	761	3045
Canoeing	7.52[b]	2.6[b]	min	349	26	68
			avg	3179	239	622
			max	20083	1510	3927
Swimming (children)	49.33[c]	1.32[d]	min	349	172	227
			avg	3179	1568	2070
			max	20083	9907	13077
Swimming (adults)	21.33[c]	1.32[d]	min	349	74	98
			avg	3179	678	895
			max	20083	4284	5654

a) Dorevitch et al. 2011
b) Rijal et al. 2011
c) Dufour et al. 2006
d) Schets et al. 2011
